# The Effect of Evolving Damage on the Finite Strain Response of Inelastic and Viscoelastic Composites

**DOI:** 10.3390/ma2041858

**Published:** 2009-11-18

**Authors:** Jacob Aboudi

**Affiliations:** Faculty of Engineering, Tel Aviv University, Ramat Aviv 69978, Israel; E-Mail: aboudi@eng.tau.ac.il

**Keywords:** large deformations, finite inelasticity, finite viscoelasticity, evolving damage, micromechanical analysis, finite strain high fidelity generalized method of cells

## Abstract

A finite strain micromechanical model is generalized in order to incorporate the effect of evolving damage in the metallic and polymeric phases of unidirectional composites. As a result, it is possible to predict the response of composites with ductile and brittle phases undergoing large coupled inelastic-damage and viscoelastic-damage deformations, respectively. For inelastic composites, both finite strain elastoplastic (time-independent) and viscoplastic (time-dependent) behaviors are considered. The ductile phase exhibits initially a hyperelastic behavior which is followed by an inelastic one, and its analysis is based on the multiplicative split of its deformation gradient into elastic and inelastic parts. The embedded damage mechanisms and their evolutions are based on Gurson’s (which is suitable for the modeling of porous materials) and Lemaitre’s finite strain models. Similarly, the polymeric phase exhibits large viscoelastic deformations in which the damage evolves according to a suitable evolution law that depends on the amount of accumulated deformation. Evolving damage in hyperelastic materials can be analyzed as a special case by neglecting the viscous effects. The micromechanical analysis is based on the homogenization technique for periodic multiphase materials, which establishes the strong form of the Lagrangian equilibrium equations. These equations are implemented together with the interfacial and periodic boundary conditions, in conjunction with the current tangent tensor of the phase. As a result, the instantaneous strain concentration tensor that relates the local deformation gradient of the phase to the externally applied deformation gradient is established. This provides also the instantaneous effective stiffness tangent tensor of the composite as well as its current response. Results are given that exhibit the effect of damage on the initial yield surfaces, response and possible failure of the composite.

## 1. Introduction

In [1], a review of finite strain micromechanical analyses of multiphase materials have been presented. It was shown that it is possible to predict the microscopic (local) and macroscopic (global) response of composites undergoing large deformations in which the constituents in these composites can be modeled as hyperelastic, thermoelastic (based on entropic elasticity), viscoelastic (including quasilinear viscoelasticity (QLV) which is suitable for the modeling of biological tissues), thermoviscoelastic, rate-dependent thermoinelastic (viscoplastic) and rate-independent thermoinelastic (elastoplastic) materials. In all cases, the micromechanical analyses were based on the homogenization technique for periodic composites, and able to provide the composite’s behavior in conjunction with the known properties of its constituents, their constitutive relations, detailed interaction and volume ratios. These analyses, referred to as *High Fidelity Generalized Method of Cells* (HFGMC), provide the instantaneous mechanical, thermal and inelastic concentration tensors that relate the local induced strain in the phase to the current externally applied strains and temperature. In addition, these micromechanical analyses yield the macroscopic constitutive equations of the multiphase composite in terms of its instantaneous stiffness (tangent) and thermal stress tensors. In anyone of these micromechanical analyses, the local field distribution among the various constituents of the composite can be also determined at any instant of loading.

Continuum damage considerations were not included in these investigation except for the prediction of the response of finite strain elastic composites where the Mullins effect can take place in the hyperelastic phase (Aboudi [2]). The purpose of the present paper is to include continuum damage mechanics considerations (see [3], [4], [5], [6], [7], for example) in finite strain elastoplastic, viscoplastic and viscoelastic materials any one of which can be a constituent in a multiphase composite. As a result, the presented generalized micromechanical analyses will enable the prediction of the behavior of composites with evolving damage in their phases.

In the case of elastoplasticity coupled with evolving damage, the model of Gurson [8] is employed as a phase in the composite. Gurson’s model is capable to describe the behavior of homogenized porous materials in which the amount of porosity corresponds to the damage variable which evolves with the applied loading from a non-zero initial value and controlled by the plastic flow. In the present paper, we adopt the two-dimensional Gurson’s model according to which the macroscopic response of a porous material is established by analyzing the field of a hollow cylinder that is subjected to a remote loading. Hence the response of the elastoplastic material in this case exhibits a transverse isotropy where the behavior in the axial direction along which the cylinder is oriented is different from the behavior in the transverse direction. The HFGMC model on the other hand can also predict the behavior of a finite strain porous elastoplastic material (which is just a special case of a composite material in which the stiffness of the second phase is negligibly small). Hence it is possible to compare the prediction of the the initial yield surfaces and response that are provided by the two independent models. Gurson’s model is also employed to represent a phase in HFGMC method. This latter modeling implies the accommodation of inelastic anisotropic constituent in a composite (microscale level) to obtain its overall inelastic anisotropic behavior (macroscale level). The considered other finite strain elastoplasticity coupled with damage model in the present investigation, is the one presented by Lemaitre [9], [10]. Here the damage variable in the material evolves with applied loading from any initial value, and the evolution is controlled by the of plastic flow rule. Both Gurson’s and Lemaitre’s models were originally formulated in the framework of small strains, and their extension to finite strain was presented by de Souza Neto *et al.* [11]. In both models, the multiplicative decomposition of the deformation gradient is employed followed by the integration of the governing equations by means of the return mapping algorithm [12], [13], in conjunction with the logarithmic strain and the backward exponential approximation (Weber and Anand [14], Eterovic and Bathe [15], Cuitino and Ortiz [16], Simo [17]).

The analysis of finite strain viscoplasticity coupled with evolving damage in a material follows the same methodology of finite elastoplasticity. Here however the consistency parameter which appears in the flow rule is not one of the unknowns but it is prescribed in advance in terms of the other unknown field variables [12] (*cf.* Perzyna’s [18]). Both the finite strain Gurson’s and Lemaitre’s models are presently extended and applied to investigate the behavior of composites that consist of viscoplastic coupled with evolving damage constituents.

A finite strain viscoelasticity theory was presented by Reese and Govindjee [19] which, in particular, allows large deviations away from the equilibrium state. This is in contrast to finite linear viscoelasticity theories, see Simo [20] and Holzapfel [21] for example and references cited in [19], which although account for large deformations, but restrict the formulation to states close to thermodynamic equilibrium by choosing linear evolution laws for the internal variables. In [19], the multiplicative split of the deformation gradient is also used, and the free energy of the viscoelastic material is expressed by the Ogden’s representation (Ogden [22], Holzapfel [21]). The integration of the viscoelastic evolution equations is performed in conjunction with the logarithmic strain and the backward exponential approximation which yield a system of nonlinear equation. Extensive comparisons between the results obtained by the theories of Reese and Govindjee [19] and Simo [20] are given by Govindjee and Reese [23]. In the finite viscoelasticity theory of Reese and Govindjee [19] damage is not taken into account. In the present investigation, evolving damage in the finite viscoelastic material is included by adopting the derivation of Lin and Schomburg [24] and Miehe and Keck [25] according to which the rate of damage depends upon the kinematic arc-length. By neglecting the viscous effects, the special case of a hyperelastic material with evolving damage is obtained (it should me noted that Gurson’s and Lemaitre’s models do not reduce to such a special case because in the absence of inelasticity, damage does not evolve).

In all cases mentioned above the constitutive equations of the finite strain inelastic and viscoelastic monolithic materials are ultimately given in terms of the first tangent tensors that relate the rates of the first Piola-Kirchhoff stress and the deformation gradient tensors. The established first tangent tensors of the various constituents are subsequently employed in the finite strain HFGMC method for the prediction of the behavior of the resulting multiphase composites.

After briefly presenting the aforementioned derivations of inelastic and viscoelastic theories coupled with evolving damage together with the corresponding computational procedures, applications are given which show the behavior of the various types of composites in various circumstances. The paper is concluded by some suggestions for further generalizations in a future research.

## 2. Finite Strain Coupled Elastoplasticity-Damage Models of Monolithic Materials

Let X and x denote the location of a point in the material with respect to the initial (Lagrangian) and current systems of coordinates, respectively, and *t* is the time. In terms of the local deformation gradient tensor F(X,t), dx=F(X,t)dX. The finite plasticity theory that is employed in the present investigation is based on the introduction of a stress-free intermediate configuration and a multiplicative decomposition of the local deformation gradient F(X,t) in the form

(1) $$\begin{array}{c} F\left(X,t\right)=F^{e}\left(X,t\right)F^{p}\left(X,t\right) \end{array}$$

where $F^{p}\left(X,t\right)$ and $F^{e}\left(X,t\right)$ are the deformation gradient tensors from the initial to the intermediate and from the intermediate to the current configuration, respectively. The corresponding right Cauchy-Green tensors are given by

(2) $$\begin{array}{c} C=F^{T}F,\;\;\;\;\;\;C^{p}=F^{pT}F^{p} \end{array}$$

where superscript *T* denotes the transpose operation. The left Cauchy-Green tensors B and $B^{e}$ are defined by

(3) $$\begin{array}{c} B=FF^{T},\;\;\;\;\;\;B^{e}=F^{e}F^{e}{}^{T} \end{array}$$

The logarithmic elastic strain $\epsilon^{e}$ is defined by

(4) $$\begin{array}{c} \epsilon^{e}=\frac{1}{2}\log\left[B^{e}\right] \end{array}$$

Two types of energy functions that model the monolithic phase in the composite are considered in the present investigation. The first one is based on the macroscopic yield function of homogenized porous materials that was established by Gurson [8]. Here, the damage variable corresponds to the amount of porosity and its evolution describes its growth with the applied loading. The second energy function is due to Lemaitre [9], [10] which includes an evolving damage variable. Both models were originally formulated in the framework of infinitesimal deformations, and their extension to finite strain analysis was presented by de Souza Neto *et al.* [11]. These models are briefly described in the following.

### 2.1. Gurson’s finite strain elastoplastic porous material model

The Hencky isotropic strain energy function per unit reference volume is defined by

(5) $$\begin{array}{c} W=\frac{1}{2}\epsilon^{e}:h:\epsilon^{e} \end{array}$$

where the fourth-order tensor h given by

(6) $$\begin{array}{c} h=\lambda I⊗I+2\mu I_{4} \end{array}$$

where *λ* and *μ* are material parameters, and I and $I_{4}$ being the identity second and fourth-order tensors, respectively. The Kirchhoff stress ***τ*** is given by

(7) $$\begin{array}{c} \tau =\frac{\partial W}{\partial \epsilon^{e}}=h:\epsilon^{e} \end{array}$$

The macroscopic yield function for homogenized porous materials that was established by Gurson [8] in which the long cylindrical pores (voids) are oriented in the 1-direction is given by

(8) $$\begin{array}{c} \Phi =\frac{1}{2}dev\;\left[\tau \right]:dev\;\left[\tau \right]-\frac{1}{3}\left[1+D^{2}-2D\cosh\left(\frac{\sqrt{3}\left(\tau_{22}+\tau_{33}\right)}{2\sigma_{y}}\right)\right]\sigma_{y}^{2} \end{array}$$

where dev[τ] is the deviator of Kirchhoff stress tensor, *D* denotes the porosity volume fraction (which represents the amount of damage) and $\sigma_{y}$ is the function that describes the hardening law of the material. For isotropic hardening it is given by

(9) $$\begin{array}{c} \sigma_{y}=Y_{0}+K\left(R\right) \end{array}$$

where $Y_{0}$ is the yield stress in simple tension and K(R) describes the isotropic hardening law. For linear hardening: K(R)=HR.

The plastic flow rule is determined from Φ by employing the relation [12]:

(10) $$\begin{array}{c} -\frac{1}{2}L_{v}\left[B^{e}\right]{\left[B^{e}\right]}^{-1}=\dot{\gamma }\frac{\partial \Phi }{\partial \tau } \end{array}$$

where $L_{v}$ is the Lie derivative of $B^{e}$ and *γ* is the consistency parameter. Here and in the following $\dot{Q}$ means the derivative of a quantity *Q* with respect to time or quasi time *t*. The Lie derivative of $B^{e}$ can be expressed in the form [13]

(11) $$\begin{array}{c} L_{v}\left[B^{e}\right]=F{\dot{C}}^{p-1}F^{T} \end{array}$$

It is worth mentioning that the flow rule (10) is identical to the one used in the book by Bonet and Wood [26], and the von Mises plasticity is readily obtained from Equation 8 by setting D=0. The finite strain plastic flow rule of Simo and Hughes [13] that was employed by Aboudi [27] however is given, apart from a numerical coefficient, by Equation 10 but with ${\left[B^{e}\right]}^{-1}$ on the left-hand-side replaced by ${\left(trace\;[B^{e}]\right)}^{-1}$. It is worth mentioning that both the present and Simo and Hughes [13] flow rules provide almost identical responses of the ductile material that is specified in the following when it is subjected to uniaxial stress loading. The responses significantly differ however for uniaxial strain loading although yielding occurs at the same strain.

The rate of hardening is determined from the relation [11]

(12) $$\begin{array}{c} \dot{R}=-\frac{\dot{\gamma }}{1-D}\frac{\partial \Phi }{\partial \sigma_{Y}} \end{array}$$

whereas the evolution law for damage (porosity) is given by

(13) $$\begin{array}{c} \dot{D}=\frac{2\dot{\gamma }}{\sqrt{3}}\left(D-D^{2}\right)\sigma_{Y}\sinh\left(\frac{\sqrt{3}\left(\tau_{22}+\tau_{33}\right)}{2\sigma_{y}}\right) \end{array}$$

Equations 10–13 together with the condition that Φ=0 are solved by employing the return mapping algorithm [11]. It should be emphasized that by means of the backward exponential approximation (Weber and Anand [14], Eterovic and Bathe [15], Cuitino and Ortiz [16], Simo [17]) the plastic flow rule that is given by Equation 10 can be reduced to the simple update expression

(14) $$\begin{array}{c} \epsilon_{n+1}^{e}=\epsilon_{n+1}^{e\;trial}-\Delta \gamma {\left.\frac{\partial \Phi }{\partial \tau }\right|}_{n+1} \end{array}$$

where $\epsilon_{n+1}^{e\;trial}$ at the present n+1 increment is computed according to Equation 4 while employing $B_{n}^{e}$ of the previous increment.

The fourth-order first tangent tensor R that is needed in the micromechanical analysis is defined by

(15) $$\begin{array}{c} \dot{T}=R\;\dot{F} \end{array}$$

where T is the first Piola-Kirchhoff stress tensor. It is related to the Kirchhoff stress tensor ***τ*** according to: $T=F^{-1}\tau$. The first tangent tensor is determined according to

(16) $$\begin{array}{ccc} R & = & \frac{\partial T}{\partial F_{n+1}}\\ & = & \frac{\partial F^{-1}}{\partial F_{n+1}}\tau +F^{-1}\frac{\partial \tau }{\partial F_{n+1}}\\ & = & \frac{\partial F^{-1}}{\partial F_{n+1}}\tau +F^{-1}\left[\frac{\partial \tau }{\partial \epsilon_{n+1}^{e\;trial}}:\frac{\partial \epsilon_{n+1}^{e\;trial}}{\partial B_{n+1}^{e\;trial}}:\frac{\partial B_{n+1}^{e\;trial}}{\partial F_{n+1}^{e\;trial}}:\frac{\partial F_{n+1}^{e\;trial}}{\partial F_{n+1}}\right] \end{array}$$

where

(17) $$\begin{array}{ccc} {\left[\frac{\partial F^{-1}}{\partial F}\right]}_{ijkl} & = & -F_{ik}^{-1}F_{lj}^{-1}\\ \frac{\partial \epsilon^{e\;trial}}{\partial B^{e\;trial}} & = & \frac{1}{2}\frac{\partial \log[B^{e\;trial}]}{\partial B^{e\;trial}}\\ {\left[\frac{\partial B^{e\;trial}}{\partial F^{e\;trial}}\right]}_{ijkl} & = & \delta_{ik}F_{jl}^{e\;trial}+\delta_{jk}F_{il}^{e\;trial}\\ {\left[\frac{\partial F^{e\;trial}}{\partial F}\right]}_{ijkl} & = & \delta_{ik}F_{lj}^{p\;-1} \end{array}$$

and $\delta_{ij}$ is the Kronecker delta. The tensor $\partial \tau /\partial \epsilon_{n+1}^{e\;trial}$ is determined by the differentiation of the set of equations that appear in the return mapping algorithm, see de Souza Neto *et al.* [11] for details.

Remark: It should be emphasized that a critical check to the validity of the established first tangent tensor R (here and in all the following cases) is that the values of the first Piola-Kirchhoff T that are obtained by the integration of Equation 15 must be identical to the values of $T=F^{-1}\tau$ that are computed directly from Equation 7 in which R is not involved.

### 2.2. Lemaitre finite strain elastoplastic damage model

The extension of Lemaitre elastoplastic model [9], [10] that includes an evolving damage to large deformations was presented by de Souza Neto *et al.* [11]. It is based on Hencky isotropic elastic-damage free energy function per unit reference volume (*cf.* Equation 5):

(18) $$\begin{array}{c} \Psi^{ed}=\frac{1}{2}\epsilon^{e}:\left(1-D\right)h:\epsilon^{e} \end{array}$$

where as before $\epsilon^{e}$ denotes the logarithmic elastic strain and *D* represents the damage variable such that 0≤D≤1. The Kirchhoff stress is determined according to, *cf.* Equation 7,

(19) $$\begin{array}{c} \tau =\frac{\partial \Psi^{ed}}{\partial \epsilon^{e}}=\left(1-D\right)h:\epsilon^{e} \end{array}$$

The thermodynamical force *Y* which is conjugate to the damage variable *D* is given by

(20) $$\begin{array}{c} Y=\frac{\partial \Psi^{ed}}{\partial D}=-\frac{1}{2}\epsilon^{e}:h:\epsilon^{e} \end{array}$$

By assuming isotropic hardening, the yield function Φ of this model is given by

(21) $$\begin{array}{c} \Phi =\frac{1}{1-D}\sqrt{3J_{2}\left(\tau \right)}-\sigma_{y} \end{array}$$

where $J_{2}\left(\tau \right)=1/2\;dev\;\left[\tau \right]:dev\;\left[\tau \right]=1/{2\;||dev\;\left[\tau \right]||}^{2}$ being the second invariant of the Kirchhoff stress deviator tensor. This yield function readily determines the plastic flow rule which is given by Equation 10. The rate of hardening is given this time, *cf.* Equation 12, by

(22) $$\begin{array}{c} \dot{R}=-\dot{\gamma }\frac{\partial \Phi }{\partial K}=\dot{\gamma } \end{array}$$

Finally, the dissipation function is taken in the form

(23) $$\begin{array}{c} \Psi =\Phi +\frac{r}{\left(1-D)(s+1\right)}{\left(\frac{-Y}{r}\right)}^{s+1} \end{array}$$

where *r* and *s* are material constants. This function provides the evolution law of damage in the form

(24) $$\begin{array}{c} \dot{D}=-\dot{\gamma }\frac{\partial \Psi }{\partial Y}=\frac{\dot{\gamma }}{1-D}{\left(\frac{-Y}{r}\right)}^{s} \end{array}$$

Equations 19–24 together with the condition that Φ=0 are solved by employing the return mapping algorithm [11]. The backward exponential approximation reduces the flow rule which is given by Equation 10 to the simple relation (14). The final constitutive relation of Lemaitre’s finite strain elastoplastic-damage model can be expressed in the form shown by Equation 15 where the fourth-order first tangent tensor R is determined by Equation 16. As in the Gurson’s model, the tensor $\partial \tau /\partial \epsilon_{n+1}^{e\;trial}$ that appears in Equation 16 is determined by the differentiation of the set of equations that appear in the return mapping algorithm.

Finally, it is worth mentioning that unlike Gurson’s model, the value of damage *D* evolves, in the framework of Lemaitre’s model, from D=0 according to Equation 24. In Gurson’s model however, a damage variable whose initial value is equal to zero does not evolve, see Equation 13.

## 3. Finite Strain Coupled Viscoplasticity-Damage Models of Monolithic Materials

The previously discussed Gurson and Lemaitre damage models were based on finite strain elastoplasticity (time-independent). Both models can be generalized to exhibit viscoplastic (time-dependent) behavior. In this situation the time derivative of the consistency parameter $\dot{\gamma }$ that appears in Equation 10 is not one of the unknowns that need to be determined as in the elastoplasticity case. Following the methodology that was presented in [12], $\dot{\gamma }$ in the coupled viscoplastic-damage Gurson’s model can be established in the form

(25) $$\begin{array}{c} \dot{\gamma }=\frac{1}{\eta }\left[{\left(\frac{3\Phi }{a\sigma_{y}^{2}}+1\right)}^{1/2\epsilon }-1\right]H\left(\Phi \right) \end{array}$$

where the yield function Φ is given by Equation 8, $a=1+D^{2}-2D\cosh\left(\frac{\sqrt{3}\left(\tau_{22}+\tau_{33}\right)}{2\sigma_{y}}\right)$, *η* and *ϵ* are parameters that characterize the viscoplastic material, and H(Φ) is the Heaviside unit step function.

For Lemaitre coupled viscoplastic-damage model, $\dot{\gamma }$ is given by

(26) $$\begin{array}{c} \dot{\gamma }=\frac{1}{\eta }\left[{\left(\frac{\Phi }{\sigma_{y}}+1\right)}^{1/\epsilon }-1\right]H\left(\Phi \right) \end{array}$$

where Φ is given by Equation 21. In the limit when η→0 and ϵ→0, the viscoplastic model recovers the perfectly plastic von Mises model [12]. It should be noted that $\dot{\gamma }$ in Perzyna’s [18] viscoplasticity model has a slightly different form from the above representation. For the Lemaitre model, for example, Equation 26 takes the form

$$\begin{array}{c} \dot{\gamma }=\frac{1}{\eta }{\left(\frac{\Phi }{\sigma_{y}}\right)}^{1/\epsilon }H\left(\Phi \right) \end{array}$$

The final constitutive law of both Gurson and Lemaitre finite strain coupled viscoplasticity-damage models has the form given by Equation 15 where the fourth-order first tangent tensor R is given by Equation 16. In this latter equation, the instantaneous fourth-order tensor $\partial \tau /\partial \epsilon_{n+1}^{e\;trial}$ is determined as follows. From Equation 14 we obtain that

(27) $$\begin{array}{c} \Delta \epsilon^{e}=\epsilon_{n+1}^{e\;trial}-\epsilon_{n}^{e}-\Delta \gamma {\left.\frac{\partial \Phi }{\partial \tau }\right|}_{n+1} \end{array}$$

It follows from Equation 7 that for Gurson’s viscoplastic model

(28) $$\begin{array}{c} \Delta \tau =h:\left(\Delta \epsilon -\Delta \epsilon^{vp}\right) \end{array}$$

where the following definitions are used

(29) $$\begin{array}{c} \Delta \epsilon \equiv \epsilon_{n+1}^{e\;trial}-\epsilon_{n}^{e},\;\;\;\;\;\;\Delta \epsilon^{vp}\equiv \Delta \gamma {\left.\frac{\partial \Phi }{\partial \tau }\right|}_{n+1} \end{array}$$

Let us multiply $h:\Delta \epsilon^{vp}$ in Equation 28 by (*cf.* Boyd and Allen [28])

(30) $$\begin{array}{c} \frac{\Delta \epsilon^{vp}:h:\Delta \epsilon }{\Delta \epsilon^{vp}:h:\Delta \epsilon }=1 \end{array}$$

This readily yields that

(31) $$\begin{array}{c} \Delta \tau =\left[h-\frac{\left(h:\Delta \epsilon^{vp}\right)⊗\left(\Delta \epsilon^{vp}:h\right)}{\Delta \epsilon^{vp}:h:\Delta \epsilon }\right]:\Delta \epsilon \end{array}$$

It follows that the requested instantaneous tangent tensor for the viscoplastic Gurson’s model is of the form

(32) $$\begin{array}{c} \frac{\partial \tau }{\partial \epsilon_{n+1}^{e\;trial}}=h-\frac{\left(h:{\dot{\epsilon }}^{vp}\right)⊗\left({\dot{\epsilon }}^{vp}:h\right)}{{\dot{\epsilon }}^{vp}:h:\dot{\epsilon }} \end{array}$$

which can be employed in Equation 16.

For Lemaitre’s viscoplastic model, Equation 19 should be employed. Hence

(33) $$\begin{array}{c} \Delta \tau =\left(1-D\right)h:\left(\Delta \epsilon -\Delta \gamma {\left.\frac{\partial \Phi }{\partial \tau }\right|}_{n+1}-\frac{\Delta D}{1-D}\epsilon_{n+1}^{e}\right) \end{array}$$

Let us denote

(34) $$\begin{array}{c} \Delta \epsilon^{vpd}\equiv \Delta \gamma {\left.\frac{\partial \Phi }{\partial \tau }\right|}_{n+1}+\frac{\Delta D}{1-D}\epsilon_{n+1}^{e} \end{array}$$

Hence the instantaneous tangent tensor for this model is given by

(35) $$\begin{array}{c} \frac{\partial \tau }{\partial \epsilon_{n+1}^{e\;trial}}=\left(1-D\right)h-\frac{\left[\left(1-D\right)h:{\dot{\epsilon }}^{vpd}\right]⊗\left[{\dot{\epsilon }}^{vpd}:\left(1-D\right)h\right]}{{\dot{\epsilon }}^{vpd}:\left(1-D\right)h:\dot{\epsilon }} \end{array}$$

which can be employed in Equation 16.

## 4. Finite Strain Coupled Viscoelasticity-Damage Model of Monolithic Materials

In the present section we briefly present the constitutive behavior of finite strain viscoelastic materials that exhibit evolving damage. The presentation follows the papers of Reese and Govindjee [19] where no damage is accounted to and Lin and Schomburg [24] where evolving damage is included. It should be mentioned that the present viscoelastic modeling allows large deviations from the thermodynamic equilibrium state and therefore is referred to as finite viscoelasticity (in contrast to finite linear viscoelasticity where small deviations from the equilibrium state is assumed).

The multiplicative decomposition of the deformation gradient is given this time by

(36) $$\begin{array}{c} F\left(X,t\right)=F^{e}\left(X,t\right)F^{v}\left(X,t\right) \end{array}$$

where $F^{v}$ is the counterpart of $F^{p}$ in Equation 1.

The modeling that is presented herein is based on a single Maxwell and elastic elements, but it can be extended to include several Maxwellian elements. The total free energy per unit reference volume is decomposed into equilibrium (EQ) which represents the strain energy of the elastic element and a nonequilibrium (NEQ) part that accounts for the Maxwell element:

(37) $$\begin{array}{c} \psi =\psi^{EQ}+\psi^{NEQ}\equiv \left(1-D\right)\psi_{0}^{EQ}+\left(1-D\right)\psi_{0}^{NEQ} \end{array}$$

where $\psi_{0}^{EQ}$ and $\psi_{0}^{NEQ}$ are referred to as the effective free energy of the undamaged material, and *D* denotes the amount of damage such that 0≤D≤1.

The resulting Kirchhoff stresses are given by

(38) $$\begin{array}{c} \tau^{EQ}=2F\frac{\partial \psi^{EQ}}{\partial C}F^{T}=\left(1-D\right)\tau_{0}^{EQ} \end{array}$$

(39) $$\begin{array}{c} \tau^{NEQ}=2F^{e}\frac{\partial \psi^{NEQ}}{\partial C^{e}}F^{e}{}^{T}=\frac{\partial \psi^{NEQ}}{\partial \epsilon^{e}}=\left(1-D\right)\tau_{0}^{NEQ} \end{array}$$

where $C^{e}=F^{e}{}^{T}F^{e}$ and $\tau_{0}^{EQ}$, $\tau_{0}^{NEQ}$ correspond to the effective Kirchhoff stresses of the undamaged material.

Let the left Cauchy-Green tensor B be represented in terms of its eigenvalues:

(40) $$\begin{array}{c} B=diag\;[b_{1},b_{2},b_{3}] \end{array}$$

With J=detF, the volume preserving tensor $\bar{B}=J^{-2/3}B$ can be accordingly represented in the form

(41) $$\begin{array}{c} \bar{B}=diag\;\left[{\bar{b}}_{1},{\bar{b}}_{2},{\bar{b}}_{3}\right]={\left(b_{1}b_{2}b_{3}\right)}^{-1/3}\;diag\;\left[b_{1},b_{2},b_{3}\right] \end{array}$$

The finite strain elastic contribution can be modeled by the Ogden’s compressible material representation (Ogden [22], Holzapfel [21]) as follows

(42) $$\begin{array}{c} \psi_{0}^{EQ}=\sum_{A=1}^{3}\omega \left({\bar{b}}_{A}\right)+\frac{K^{e}}{2}{\left(J-1\right)}^{2} \end{array}$$

where $K^{e}$ is the elastic bulk modulus and

(43) $$\begin{array}{c} \omega \left({\bar{b}}_{A}\right)=\sum_{r=1}^{N}\frac{\mu_{r}^{e}}{\alpha_{r}^{e}}{\left({\bar{b}}_{A}\right)}^{\alpha_{r}^{e}/2} \end{array}$$

and $\mu_{r}^{e}$ and $\alpha_{r}^{e}$ are material parameters of the elastic element.

For Maxwell’s element, the free energy is represented by [19]:

(44) $$\begin{array}{c} \psi_{0}^{NEQ}=\sum_{p=1}^{3}\frac{\mu_{p}^{v}}{\alpha_{p}^{v}}\left[{\left({\bar{b}}_{1}^{e}\right)}^{\alpha_{p}^{v}/2}+{\left({\bar{b}}_{2}^{e}\right)}^{\alpha_{p}^{v}/2}+{\left({\bar{b}}_{3}^{e}\right)}^{\alpha_{p}^{v}/2}-3\right]+\frac{K^{v}}{4}\left[{\left(J^{e}\right)}^{2}-2\log J^{e}-1\right] \end{array}$$

where

(45) $$\begin{array}{c} B^{e}=diag\;\left[b_{1}^{e},b_{2}^{e},b_{3}^{e}\right] \end{array}$$

and $J^{e}=\sqrt{b_{1}^{e}b_{2}^{e}b_{3}^{e}}$, ${\bar{b}}_{A}^{e}={\left(J^{e}\right)}^{-2/3}b_{A}^{e}$, and $\mu_{p}^{v}$, $\alpha_{p}^{v}$, $K^{v}$ are material parameters. By employing Eqs. (37) and (39) the following expression for the principal values of $\tau_{0}^{NEQ}$ is obtained

(46) $$\begin{array}{c} \tau_{0A}^{NEQ}=\sum_{p=1}^{3}\mu_{p}^{v}\left[\frac{2}{3}{\left({\bar{b}}_{A}^{e}\right)}^{\alpha_{p}^{v}/2}-\frac{1}{3}{\left({\bar{b}}_{B}^{e}\right)}^{\alpha_{p}^{v}/2}-\frac{1}{3}{\left({\bar{b}}_{C}^{e}\right)}^{\alpha_{p}^{v}/2}\right]+\frac{K^{v}}{2}\left[{\left(J^{e}\right)}^{2}-1\right],\;\;\;\;\;\;A,B,C=1,2,3 \end{array}$$

The evolution equation in the present finite viscoelastic case that replaces Equation 10 is given by ([19])

(47) $$\begin{array}{c} -\frac{1}{2}L_{v}\left[B^{e}\right]{\left[B^{e}\right]}^{-1}=\frac{1}{2\eta_{D}}dev\;\left[\tau^{NEQ}\right]+\frac{1}{9\eta_{V}}trace\;\left[\tau^{NEQ}\right] \end{array}$$

where $\eta_{D}$ and $\eta_{V}$ are the deviatoric and volumetric viscosities, respectively, and presently (*cf.* Equation 11)

(48) $$\begin{array}{c} L_{v}\left[B^{e}\right]=F{\dot{C}}^{v-1}F^{T} \end{array}$$

with $C^{v}=F^{vT}F^{v}$. For elastic bulk behavior, $1/\eta_{V}=0$.

By employing the exponential mapping algorithm, Equation 47 is reduced to, *cf.* Equation 14,

(49) $$\begin{array}{c} \epsilon_{n+1,A}^{e}=\epsilon_{n+1,A}^{e\;trial}-\Delta t{\left[\frac{1}{2\eta_{D}}dev\;\left[\tau_{A}^{NEQ}\right]+\frac{1}{9\eta_{V}}trace\;\left[\tau^{NEQ}\right]\right]}_{n+1} \end{array}$$

where the principal values of the logarithmic strain $\epsilon_{A}^{e}$ are given by, *cf.* Equation 4, $\epsilon_{A}^{e}=1/2\log\left(b_{A}^{e}\right)$ and Δt is the time increment between the current and previous step. Equation 49 forms a system of coupled nonlinear equations in the three unknowns: $\epsilon_{A}^{e}$, A=1,2,3. It readily provides, *cf.* Equation 27, that

(50) $$\begin{array}{c} \Delta \epsilon_{A}^{e}=\epsilon_{n+1,A}^{e\;trial}-\epsilon_{n,A}^{e}-\Delta t{\left[\frac{1}{2\eta_{D}}dev\;\left[\tau_{A}^{NEQ}\right]+\frac{1}{9\eta_{V}}trace\;\left[\tau^{NEQ}\right]\right]}_{n+1} \end{array}$$

The rate of damage evolution is given by Lin and Schomburg [24] and Miehe and Keck [25] in the form

(51) $$\begin{array}{c} \dot{D}=\frac{\dot{z}}{\eta_{dam}}\left(D^{\infty }-D\right) \end{array}$$

where

(52) $$\begin{array}{c} \dot{z}=\sqrt{\frac{2}{3}}\left|\right|\dot{H}\left|\right|,\;\;\;\;\;\;H=\frac{1}{2}\log C \end{array}$$

with the saturation value

(53) $$\begin{array}{c} D^{\infty }=\frac{1}{1+D_{0}^{\infty }\exp\left(-\beta_{dam}/\alpha_{dam}\right)} \end{array}$$

and

(54) $$\begin{array}{c} \beta_{dam}=\max_{0\leq \xi \leq t}\sqrt{\frac{2}{3}}\left||H(\xi )|\right| \end{array}$$

In these relations, $\eta_{dam}$, $D_{0}^{\infty }$ and $\alpha_{dam}$ are material parameters.

The fourth-order first tangent tensor $R^{NEQ}$ is determined by employing Equation 16. In this equation the expression $\partial \tau^{NEQ}/\partial \epsilon_{n+1}^{e\;trial}$ is determined as follows. From Equation 39 the following expression can be established

(55) $$\begin{array}{c} \Delta \tau_{A}^{NEQ}=\left(1-D\right)\Delta \tau_{0A}^{NEQ}-\tau_{0A}^{NEQ}\Delta D \end{array}$$

Let the second-order tensor M be defined by

(56) $$\begin{array}{c} M=\left[M_{AB}\right]\equiv \left[\frac{\partial \tau_{0A}^{NEQ}}{\partial \epsilon_{B}^{e}}\right],\;\;\;\;\;\;A,B=1,2,3 \end{array}$$

With

(57) $$\begin{array}{c} \tau_{0A}^{NEQ}=dev\;\left[\tau_{0A}^{NEQ}\right]+\frac{1}{3}trace\;\left[\tau_{0}^{NEQ}\right] \end{array}$$

the explicit components of M are given by [19]:

(58) $$\begin{array}{c} \frac{\partial dev\;[\tau_{0A}^{NEQ}]}{\partial \epsilon_{A}^{e}}=\sum_{p=1}^{3}\mu_{p}^{v}\alpha_{p}^{v}\left[\frac{4}{9}{\left({\bar{b}}_{A}^{e}\right)}^{\alpha_{p}^{v}/2}+\frac{1}{9}{\left({\bar{b}}_{B}^{e}\right)}^{\alpha_{p}^{v}/2}+\frac{1}{9}{\left({\bar{b}}_{C}^{e}\right)}^{\alpha_{p}^{v}/2}\right] \end{array}$$

(59) $$\begin{array}{c} \frac{\partial dev\;[\tau_{0A}^{NEQ}]}{\partial \epsilon_{B}^{e}}=\sum_{p=1}^{3}\mu_{p}^{v}\alpha_{p}^{v}\left[-\frac{2}{9}{\left({\bar{b}}_{A}^{e}\right)}^{\alpha_{p}^{v}/2}-\frac{2}{9}{\left({\bar{b}}_{B}^{e}\right)}^{\alpha_{p}^{v}/2}+\frac{1}{9}{\left({\bar{b}}_{C}^{e}\right)}^{\alpha_{p}^{v}/2}\right] \end{array}$$

and

(60) $$\begin{array}{c} \frac{\partial trace\;[\tau_{0}^{NEQ}]}{\partial \epsilon_{A}^{e}}=3K^{v}{\left(J^{e}\right)}^{2} \end{array}$$

In conjunction with Equation 50, we obtain that

(61) $$\begin{array}{ccc} \Delta \tau_{A}^{NEQ} & = & \left(1-D\right)M_{AB}\left\{\epsilon_{n+1,B}^{e\;trial}-\epsilon_{n,B}^{e}-\Delta t\left[{\left.\frac{1}{2\eta_{D}}dev\;\left[\tau_{B}^{NEQ}\right]+\frac{1}{9\eta_{V}}trace\;\left[\tau^{NEQ}\right]\right]}_{n+1}\right.\right\}\\ & - & \tau_{0A}^{NEQ}\Delta D \end{array}$$

Hence

(62) $$\begin{array}{ccc} \Delta \tau_{A}^{NEQ} & = & \left(1-D\right)M_{AB}\{\epsilon_{n+1,B}^{e\;trial}-\epsilon_{n,B}^{e}-\Delta t{\left[\frac{1}{2\eta_{D}}dev\;\left[\tau_{B}^{NEQ}\right]+\frac{1}{9\eta_{V}}trace\;\left[\tau^{NEQ}\right]\right]}_{n+1}\\ & - & M_{BQ}^{-1}\tau_{0Q}^{NEQ}\frac{\Delta D}{1-D}\} \end{array}$$

Let $\Delta \epsilon_{A}^{e}$ and $\Delta \epsilon_{A}^{ved}$ be defined by

(63) $$\begin{array}{ccc} \Delta \epsilon_{A} & \equiv & \epsilon_{n+1,A}^{e\;trial}-\epsilon_{n,A}^{e}\\ \Delta \epsilon_{A}^{ved} & \equiv & \Delta t{\left[\frac{1}{2\eta_{D}}dev\;\left[\tau_{A}^{NEQ}\right]+\frac{1}{9\eta_{V}}trace\;\left[\tau^{NEQ}\right]\right]}_{n+1}+M_{AB}^{-1}\tau_{0B}^{NEQ}\frac{\Delta D}{1-D} \end{array}$$

Therefore Equation 62 can be represented by

(64) $$\begin{array}{c} \Delta \tau_{A}^{NEQ}=\left(1-D\right)M_{AB}\left[\Delta \epsilon_{B}-\Delta \epsilon_{B}^{ved}\right] \end{array}$$

By following the previous derivation for the establishment of the tangent tensor in the viscoplastic case, the following expression can be obtained

(65) $$\begin{array}{c} \frac{\partial \tau_{A}^{NEQ}}{\partial \epsilon_{B}^{e\;trial}}=\left(1-D\right)M-\frac{\left[\left(1-D\right)M:{\dot{\epsilon }}^{ved}\right]⊗\left[{\dot{\epsilon }}^{ved}:\left(1-D\right)M\right]}{{\dot{\epsilon }}^{ved}:\left(1-D\right)M:\dot{\epsilon }} \end{array}$$

With $\tau_{A}^{NEQ}$, A=1,2,3, and their derivatives given by Equations 39, 46 and 65, the derivative fourth-order tensor function $\partial \tau^{NEQ}/\partial \epsilon_{n+1}^{e\;trial}$ can be established by employing the explicit expressions given by [11]. Similar treatment applies to $\tau^{EQ}$ which leads to the establishment of $\partial \tau^{EQ}/\partial \epsilon_{n+1}^{e}$ and $R^{EQ}$. The first tangent tensor of the viscoelastic material is given by $R=R^{NEQ}+R^{EQ}$. The special case of a hyperelastic material coupled with evolving damage can be obtained by disregarding the viscous effects so that $\psi^{NEQ}=0$.

## 5. Finite Strain Micromechanical Analysis

Finite strain HFGMC micromechanical analyses for the establishment of the macroscopic constitutive equations of various types of composites with doubly periodic microstructure have been previously reviewed by Aboudi [1]. These micromechanical analyses are based on the homogenization technique in which a repeating unit cell of the periodic composite can identified. This repeating unit cell represents the periodic composite in which the double periodicity is taken in the transverse 2−3 plane, so that the axial 1-direction corresponds to the continuous direction (for a fiber-reinforced material, for example, the 1-direction coincides with the fibers orientation). In the framework of these HFGMC micromechanical models, the displacements are asymptotically expanded and the repeating unit cell is discretized. This is followed by imposing the equilibrium equations, the displacement and traction interfacial conditions as well as the conditions that ensure that the displacements and tractions are periodic across the repeating unit cell. In particular, the imposition of the equilibrium equations provide the strong form of the Lagrangian equilibrium conditions of the homogenization theory that must be satisfied. The resulting homogenization derivation establishes the deformation concentration tensor A(Y), where Y are the local Lagrangian system of coordinates with respect to which field variables in the repeating unit cell are characterized. This tensor relates the rate of the local deformation gradient gradient $\dot{F}\left(Y\right)$ at a material point Y within the repeating unit cell to the externally applied deformation gradient rate $\dot{\bar{F}}$ in the form:

(66) $$\dot{F}\left(Y\right)=A\left(Y\right):\dot{\bar{F}}$$

It follows from Equation 66 and in conjunction with (15) that the local stress rate at this point is given by

(67) $$\dot{T}\left(Y\right)=R\left(Y\right):\left(A\left(Y\right):\dot{\bar{F}}\right)$$

Hence the resulting macroscopic constitutive equation for the multiphase composite undergoing large deformation is given by

(68) $$\dot{\bar{T}}=R^{*}:\dot{\bar{F}}$$

where $R^{*}$ is the instantaneous effective stiffness (first tangent) tensor of the multiphase composite. It can be expressed in terms of the first tangent tensors of the constituents R(Y) and the established deformation concentration tensor A(Y) in the form

(69) $$R^{*}=\frac{1}{V_{Y}}\int_{V_{Y}}R\left(Y\right)\;A\left(Y\right)dV_{Y}$$

where $V_{Y}$ is the volume of the repeating unit cell. More details can be found in the aforementioned reference [1]. It should be noted that the current value of $R^{*}$ of the composite is affected by the current value of damage variable through the instantaneous value of tangent tensors R(Y) of the finite strain constituents.

The finite strain HFGMC micromechanical model predictions were assessed verified by comparison with analytical and numerical large deformation solutions by Aboudi and Pindera [29], Aboudi [27] and Aboudi [2] for elastic, elastoplastic and elastic composite in which the Mullins damage effect is incorporated with the hyperelastic constituents, respectively.

## 6. Computational Procedures

In the following, the computational procedures for the determination of the finite strain response of the inelastic and viscoelastic composites are described.

### 6.1. Finite strain inelastic composites

At time $t_{n}$, the local deformation gradient tensor $F_{n}$ and the elastic left Cauchy-Green tensor $B_{n}^{e}$ have been already established in the inelastic phase of the composite. In addition, $F_{n+1}$ is assumed to be known at time $t_{n+1}=t_{n}+\Delta t$.

(1) Compute the local trial logarithmic strain $\epsilon_{n+1}^{e\;trial}$ as follows:

(70) $$\begin{array}{ccc} f_{n+1} & = & F_{n+1}F_{n}^{-1}\\ B_{n+1}^{e\;trial} & = & f_{n+1}B_{n}^{e}f_{n+1}^{T}\\ \epsilon_{n+1}^{e\;trial} & = & \frac{1}{2}log\left[B_{n+1}^{e\;trial}\right] \end{array}$$

(2) With $\epsilon_{n+1}^{e\;trial}$, compute $\tau_{n+1}^{trial}$ from Equation 7 and solve the nonlinear system of equations that consists of Equation 14, the appropriate yield condition of the elastoplastic constituent, the evolution equations of the internal and damage variables in the constituent. In the case of a composite with a viscoplastic phase, the yield conditions of the constituent are not part of the system and, as previously mentioned, $\dot{\gamma }$ is not one of the unknowns. The solution of this system of nonlinear equations provides the current variables in the inelastic constituent of the composite at time step n+1.

(3) The instantaneous first tangent tensor R of the constituent can be determined from Equation 16.

(4) With the established local value of R in the inelastic phase, it is possible to proceed with the micromechanical analysis and compute the current concentration tensor A that appears in Equation 66, see [1]. Consequently, Equation 69 can be employed to determine the instantaneous effective tangent tensor $R^{*}$ of the composite.

(5) The rate of the externally applied deformation gradient $\dot{\bar{F}}$ is determined in accordance with the prescribed type of loading. For a uniaxial deformation in the 1-direction, for example, ${\dot{\bar{F}}}_{11}$ is known while all other components of $\dot{\bar{F}}$ are zero. Hence, it is possible to compute the stress rate $\dot{\bar{T}}$ using Equation 68. In addition, by integrating the resulting global stress rate, $\bar{T}$ is obtained.

(6) Once $\dot{\bar{F}}$ has been determined, it is possible to compute the rates of the local deformation gradients from Equation 66. The integration of the latter would yield the local deformation gradients in the constituent to be used in the next time step.

(7) If, on the other hand, a uniaxial stress loading is applied on the composite such that ${\dot{\bar{F}}}_{11}$ only is known together with $\bar{T}$ the components of which are zero in the other directions, an iterative procedure is needed to determine the other components of $\dot{\bar{F}}$ from these conditions.

### 6.2. Finite strain viscoelastic composites

At time $t_{n}$, the local deformation gradient $F_{n}$ and the left Cauchy-Green deformation tensors $B_{n}^{e}$ have been already established, and $F_{n+1}$ is assumed to be known.

(1) From $F_{n}$ and $F_{n-1}$, the local right Cauchy-Green deformation tensors $C_{n}$ and $C_{n-1}$ can be readily determined. Hence $\dot{z}$ in Equation 52 can be determined, from which $\dot{D}$ that is given by Equation 51 can be integrated to provide the damage variable at time $t_{n+1}$:

(71) $$\begin{array}{c} D_{n+1}=\frac{\Delta zD^{\infty }+\eta_{dam}D_{n}}{\Delta z+\eta_{dam}} \end{array}$$

Equation 54 yields

(72) $$\beta_{dam}^{n+1}=\left\{\begin{array}{cc} \sqrt{\frac{2}{3}}\left|\right|H_{n+1}\left|\right|, & if\;\sqrt{\frac{2}{3}}\left|\right|H_{n+1}\left|\right|-\beta_{dam}^{n}>0\\ \sqrt{\frac{2}{3}}\left|\right|H_{n}\left|\right|, & \mathrm{otherwise} \end{array}\right.$$

(2) The local trial elastic left Cauchy-Green deformation tensor $B_{n+1}^{e\;trial}$ can be computed from the second relation in Equation 70.

(3) From $B_{n+1}^{e\;trial}$ the principal values $b_{A}^{e\;trial}$ and the logarithmic strains $\epsilon_{A}^{e\;trial}$ can be determined at time step n+1. The solution of the nonlinear equations (50) provides $b_{A}^{e}$, $\epsilon_{A}^{e}$ and $B^{e}$ at time step n+1.

(4) From the established $b_{A}^{e}$, the Kirchhoff stresses $\tau_{A}$ and tensor M can be calculated from Equations 46 and 56, respectively.

(5) The local tangent tensors $R^{NEQ}$ and $R^{EQ}$ are determined from the procedure that leads to Equation 16 in conjunction with Equation 65.

(6) With the established local values of $R=R^{NEQ}+R^{EQ}$ of the constituent, it is possible to proceed with the micromechanical analysis to compute the concentration tensor A that appears in Equation 66, see [1]. Consequently, Equation 69 can be employed to determine the tangent tensor $R^{*}$.

(7) The rate of the externally applied deformation gradient $\dot{\bar{F}}$ is determined in accordance with the prescribed type of loading. For a uniaxial deformation in the 1-direction, for example, ${\dot{\bar{F}}}_{11}$ is known while all other components of $\dot{\bar{F}}$ are zero. Hence, it is possible to compute the stress rate $\dot{\bar{T}}$ using Equation 68. In addition, by integrating the resulting global stress rate, $\bar{T}$ is obtained.

(8) Once $\dot{\bar{F}}$ has been determined, it is possible to compute the rates of the local deformation gradients from Equation 66. The integration of the latter would yield the local deformation gradients to be used in the next time step.

(9) If, on the other hand, a uniaxial stress loading is applied on the composite such that ${\dot{\bar{F}}}_{11}$ only is known together with $\bar{T}$ the components of which are zero in the other directions, an iterative procedure is needed to determine the other components of $\dot{\bar{F}}$ from these conditions.

## 7. Applications

In this section, applications are given for the various models with evolving damage. For the inelastic constituent we consider a ductile material with linear hardening whose properties are given in Table 1 (In the range of small deformations these parameters correspond to the characterization of the aluminum alloy 2024-T4). The parameters of the finite viscoelastic material, Equation 44, are given in Table 2 together with $\eta_{D}$ and $1/\eta_{V}=0$ (assuming elastic bulk deformations).

**Table 1:** Material parameters of the ductile material.

*E* (GPa)	*ν* (GPa)	$Y_{0}$ (MPa)	*H* (GPa)
72.4	0.33	286.67	11.7

The parameters *E*, *ν*, $Y_{0}$ and *H* denote the Young’s modulus, Poisson’s ratio, yield stress in simple tension and linear hardening slope.

**Table 2:** Material parameters of the viscoelastic material (Reese and Govindjee [19]).

$\mu_{1}^{v}$(MPa)	$\mu_{2}^{v}$(MPa)	$\mu_{3}^{v}$(MPa)	$\alpha_{1}^{v}$	$\alpha_{2}^{v}$	$\alpha_{3}^{v}$	$K^{v}$(MPa)	$\eta_{D}$(MPa s)
0.3544	−0.124	0.0266	1.8	−2	7	50	9.38105

The parameters $\mu_{p}^{v}$ and $\alpha_{p}^{v}$, p=1,2,2 are the Ogden’s material constants, $K^{v}$ is the bulk modulus and $\eta_{D}$ is the viscoelastic constant of the viscous element with $1/\eta_{v}=0$.

### 7.1. Application of the finite strain Gurson’s coupled elastoplastic-damage model

Gurson’s coupled elastoplastic-damage two-dimensional model that was previously presented corresponds to the modeling of the effective behavior of a porous material in which the long cylindrical pores are oriented in the 1-direction. Consequently, it should be interesting to compare the predictions of the Gurson’s model with the corresponding ones obtained from HFGMC micromechanical analysis. To this end, the doubly periodic HFGMC in which one of the phases has zero stiffness is considered. the volume fraction of this phase is taken to be equal to the porosity *D* in Equation 8. We start our investigation by comparing the initial yield surfaces as predicted by the Gurson’s and HFGMC models.

For the Gurson’s model the initial yielding is determined from Equation 8 which provides

(73) $$\frac{3}{2}dev\;\left[\tau \right]:dev\;\left[\tau \right]=\left[1+D^{2}-2D\cosh\left(\frac{\sqrt{3}\left(\tau_{22}+\tau_{33}\right)}{2Y_{0}}\right)\right]Y_{0}^{2}$$

The von Mises yielding criterion is obtained from this equation by setting D=0. Suppose that in the Gurson’s model the initial yield surface ${\bar{T}}_{22}$ against ${\bar{T}}_{33}$ is requested, where $\bar{T}=F^{-1}\tau$ being the first Piola-Kirchhoff stress tensor. Here all components ${\bar{T}}_{ij}$ are equal to zero except ${\bar{T}}_{22}$ and ${\bar{T}}_{33}$ that may take any combination. Hence let us represent these components in the form

(74) $${\bar{T}}_{22}=A\cos\theta ,\;\;\;\;\;\;{\bar{T}}_{33}=A\sin\theta$$

where *A* is the radial distance of a point located on the initial yield surface envelope ${\bar{T}}_{22}$-${\bar{T}}_{33}$ and *θ* is the corresponding polar angle. The substitution of these values in Equation 73, shows that for a given angle *θ*, the radial distance of $A/Y_{0}$ of this point is given by root of the transcendental equation

(75) $$\frac{A}{Y_{0}}=\sqrt{\frac{1+D^{2}-2D\cosh\left(\frac{\sqrt{3}A\left(F_{22}\cos\theta +F_{33}\sin\theta \right)}{2Y_{0}}\right)}{F_{22}\cos^{2}\theta +F_{33}\sin^{2}\theta -F_{22}F_{33}\sin\theta \cos\theta }}$$

where in Equation 73, $\tau_{22}=F_{22}{\bar{T}}_{22}$ and $\tau_{33}=F_{33}{\bar{T}}_{33}$ have been substituted. Since in typical metallic materials yielding takes place at very small strains (in aluminum, for example, yielding in simple tension accrues at a strain of about 0.4 percent), it can be practically assumed that $F_{22}=F_{33}\approx 1$. Other types of initial yield surfaces can be generated in the same manner.

The initial yield surfaces that are predicted by the HFGMC model, on the other hand, can be generated by establishing the instantaneous stress concentration tensor B(Y) which relates the rate of the local Piola-Kirchhoff stress tensor $\dot{T}\left(Y\right)$ to the externally applied stress rate $\dot{\bar{T}}$, namely

(76) $$\dot{T}\left(Y\right)=B\left(Y\right):\dot{\bar{T}}$$

From Equations 66–68 this tensor can be shown to be given by

(77) $$B\left(Y\right)=R\left(Y\right)\;A\left(Y\right)\;{\left[R^{*}\right]}^{-1}$$

Corresponding to the above discussion, suppose that the initial yield envelope ${\bar{T}}_{22}$ against ${\bar{T}}_{33}$ is requested. Here too all the average (composite) Piola-Kirchhoff stress components $\bar{T}$ are equal to zero except ${\bar{T}}_{22}$ and ${\bar{T}}_{33}$ which can be represented by Equation 74. The initial yielding of any ductile phase of the HFGMC model is given by the von Mises criterion namely, it is given by Equation 73 in which D=0 is substituted. In addition, for initial yielding at very small strains, Equation 76 can be reduced to $T=B\bar{T}$. Hence the initial yielding of a point whose polar angle is *θ* as predicted by the HFGMC is given by

(78) $$\frac{A}{Y_{0}}=\min_{over\;all\;HFGMC\;ductile\;phases}\sqrt{\frac{1}{B_{22}\cos^{2}\theta +B_{33}\sin^{2}\theta -B_{22}B_{33}\sin\theta \cos\theta }}$$

**Figure 1:**
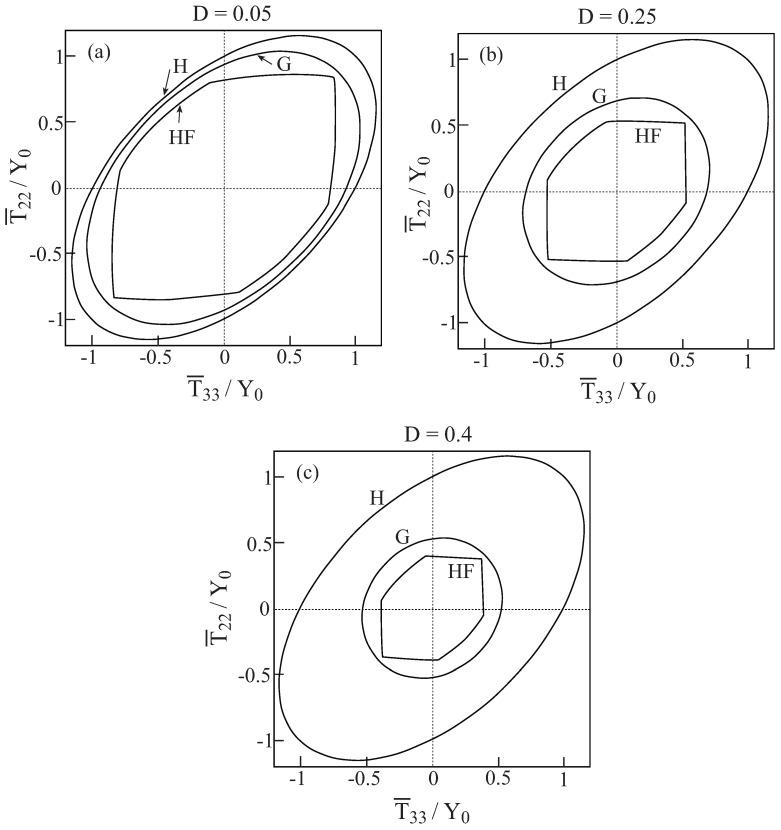
Comparisons between the initial yield surfaces ${\bar{T}}_{22}-{\bar{T}}_{33}$ as predicted by the Gurson’s (G) and HFGMC (HF) models. Also shown, as a reference, is the simple von Mises envelope in a homogeneous elastoplastic material (H). (a) D=0.05, (b) D=0.25, (c) D=0.4.

**Figure 2:**
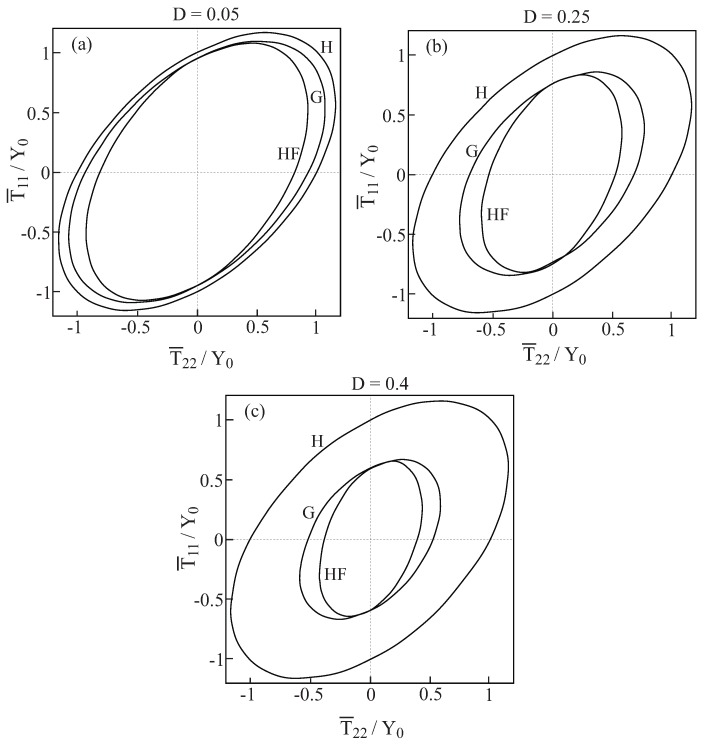
Comparisons between the initial yield surfaces ${\bar{T}}_{11}-{\bar{T}}_{22}$ as predicted by the Gurson’s (G) and HFGMC (HF) models. Also shown, as a reference, is the simple von Mises envelope in a homogeneous elastoplastic material (H). (a) D=0.05, (b) D=0.25, (c) D=0.4.

**Figure 3:**
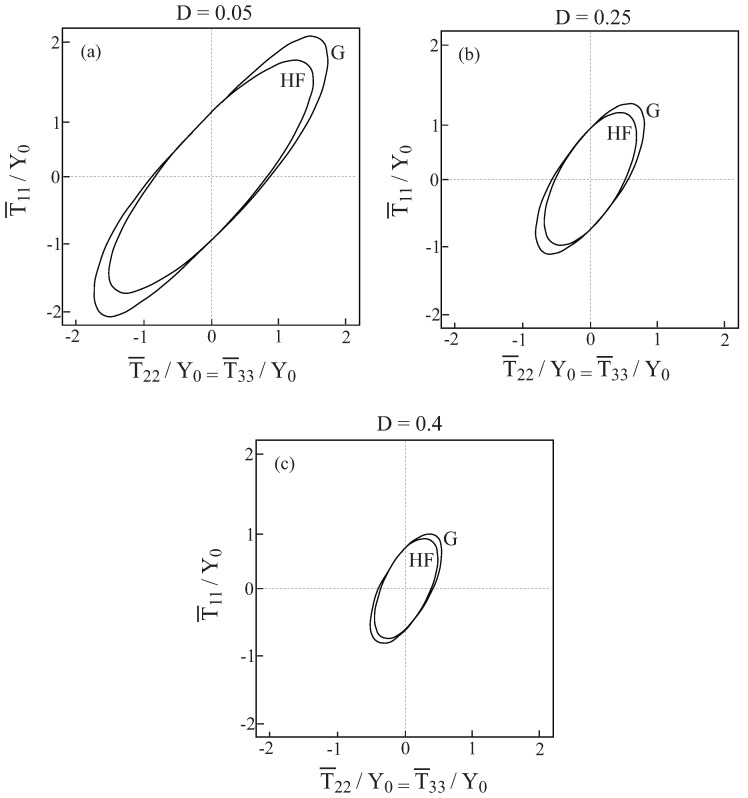
Comparisons between the initial yield surfaces ${\bar{T}}_{11}-{\bar{T}}_{22}={\bar{T}}_{33}$ as predicted by the Gurson’s (G) and HFGMC (HF) models. (a) D=0.05, (b) D=0.25, (c) D=0.4.

In Figure 1, comparisons between the initial yield surfaces ${\bar{T}}_{22}/Y_{0}$ against ${\bar{T}}_{33}/Y_{0}$ as predicted by the Gurson’s and HFGMC models are shown for three values of damage (porosity) values: D=0.05,0.25 and 0.4. Also shown as a reference is the simple von Mises criterion of a homogeneous elastoplastic material namely: $\sqrt{\frac{3}{2}dev\;\left[\tau \right]:dev\;\left[\tau \right]}=Y_{0}$. It can be clearly observed that a fair correspondence between the two models exist. Next, Figure 2 shows the initial yield surfaces in the ${\bar{T}}_{11}$-${\bar{T}}_{22}$ plane. Since the voids in the Gurson and HFGMC models are oriented in the 1-direction, the symmetry which can be observed in Figure 1 in the 2 and 3-directions does not exist anymore in the 1 and 2-direction in Figure 2. Here too, fair agreement between the two models can be observed. Finally, let us examine the initial yield envelopes for the loading ${\bar{T}}_{11}$ and ${\bar{T}}_{22}={\bar{T}}_{33}$. The resulting envelopes are shown in Figure 3 for the above three values of porosity. It should be noted that the simple von Mises criterion (for yielding of homogeneous ductile materials) reduces in this case to two parallel straight lines ${\bar{T}}_{11}-{\bar{T}}_{22}=\pm Y_{0}$ the first one of which passes the points: $\left({\bar{T}}_{11}/Y_{0}=1,{\bar{T}}_{22}/Y_{0}=0\right)$ and $\left({\bar{T}}_{11}/Y_{0}=0,{\bar{T}}_{22}/Y_{0}=-1\right)$, whereas the second one passes through the points: $\left({\bar{T}}_{11}/Y_{0}=-1,{\bar{T}}_{22}/Y_{0}=0\right)$ and $\left({\bar{T}}_{11}/Y_{0}=0,{\bar{T}}_{22}/Y_{0}=1\right)$, with the expected result that yielding will not occur for stress values between these two lines. Here too, the correspondence between the two models is quite good. In conclusion, these three figures show that the simple Gurson’s model is quite reliable for the prediction of initial yield surfaces of porous materials.

**Figure 4:**
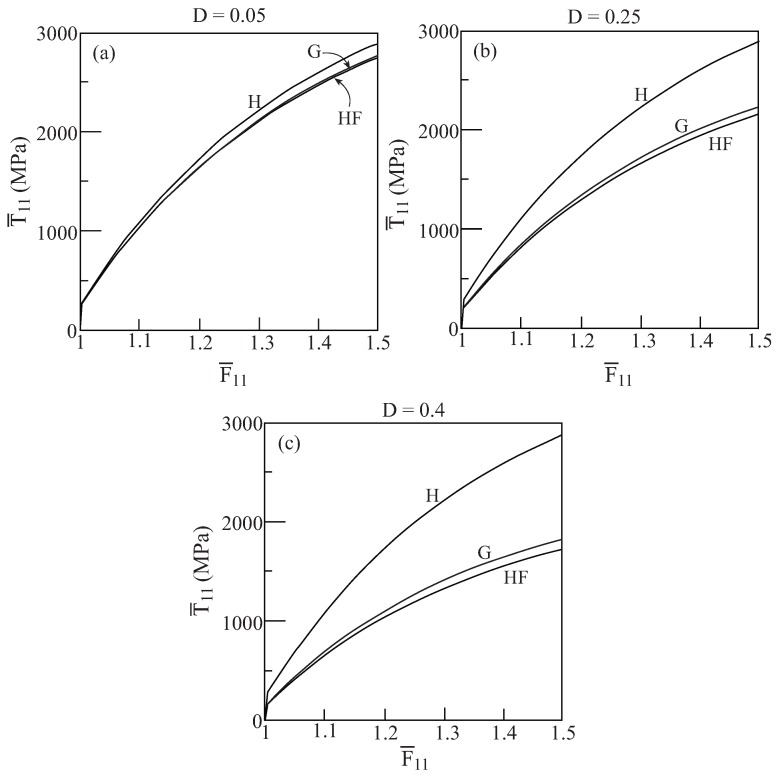
Comparisons between the uniaxial stress response to loading in the axial 1-direction of the porous ductile elastoplastic material whose matrix properties are given in Table 1 as predicted by the Gurson (G) and HFGMC (HF) models. Also shown for a reference is the uniaxial stress response of the homogeneous elastoplastic material (H). (a) D=0.05, (b) D=0.25, (c) D=0.4.

Thus far the initial yield envelopes that are predicted by the two models have been investigated. The next investigation is concerned with the responses of porous materials that are obtained by the Gurson and HFGMC model. To this end, let us consider the ductile material that is characterized by Table 1. The response of this material with three values of porosity: D=0.05, 0.25 and 0.4, is shown in Figure 4. This figure shows the average uniaxial stress responses ${\bar{T}}_{11}-{\bar{F}}_{11}$ to loading in the 1-direction (*i.e.*, in the direction of which the pores are oriented) as predicted by the Gurson and HFGMC model. Also shown for reference is the uniaxial stress response of the bulk material (*i.e.*, with zero porosity D=0). The graphs show that good agreement between the predictions of Gurson and HFGMC model exists. The effect of porosity (damage) is clearly exhibited by comparison with the homogeneous material behavior.

**Figure 5:**
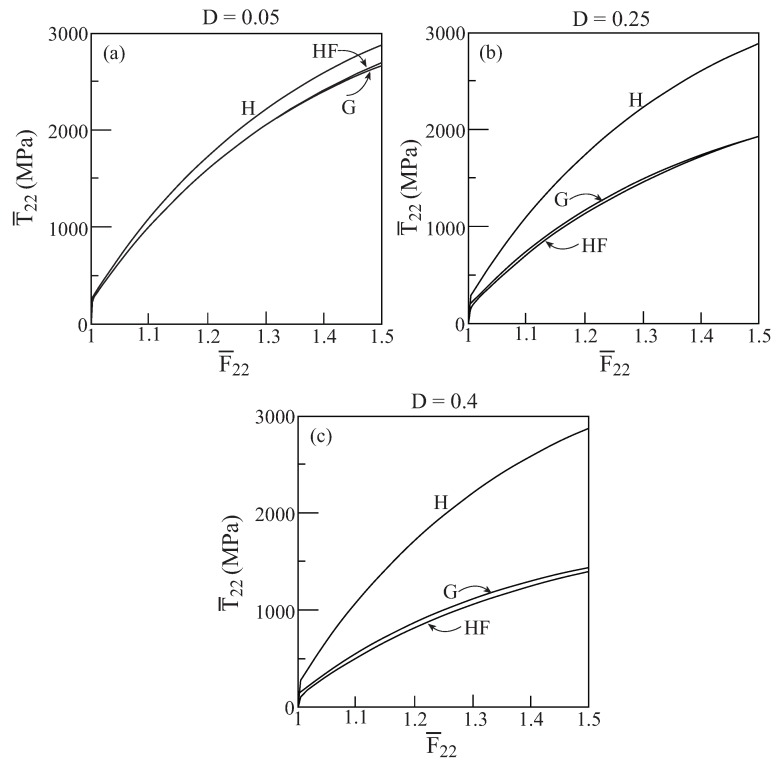
Comparisons between the uniaxial stress response to loading in the transverse 2-direction of the porous ductile elastoplastic material whose matrix properties are given in Table 1 as predicted by the Gurson (G) and HFGMC (HF) models. Also shown for a reference is the uniaxial stress response of the elastoplastic homogeneous material (H). (a) D=0.05, (b) D=0.25, (c) D=0.4.

The responses ${\bar{T}}_{22}-{\bar{F}}_{22}$ as predicted by the two models to a uniaxial stress loading of the porous material in the transverse 2-direction are shown in Figure 5 for three values of porosity (the response of the homogeneous material is included for comparison). Here too, very good agreement between the Gurson and HFGMC model can be clearly observed. It should be noted that a careful comparison between the flow stress levels of Figure 4 and Figure 5 reveals that the axial 1-direction along which the pore extends is relatively stronger (exhibiting higher stresses) than the transverse 2-direction. This observation is consistent with Figure 2 which shows that the porous material yields earlier when loaded in the transverse direction as compared to a loading in the axial direction.

**Figure 6:**
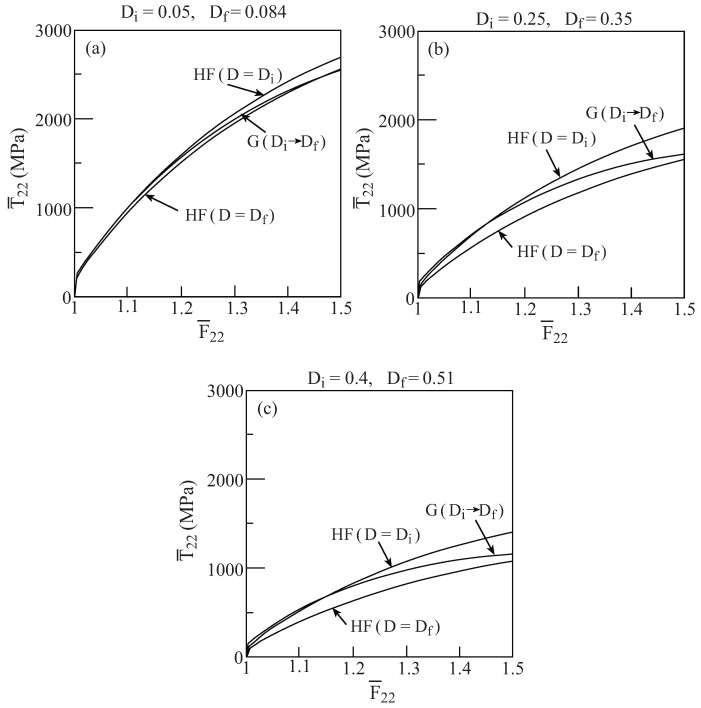
Comparisons between the uniaxial stress response to loading in the transverse 2-direction of the porous ductile elastoplastic material whose matrix properties are given in Table 1 as predicted by the Gurson and HFGMC models. In the Gurson’s elastoplastic model, the initial porosity (damage) is $D_{i}$ which subsequently evolves to the final value $D_{f}$ when ${\bar{F}}_{22}=1.5$. The corresponding responses that are predicted by the HFGMC model are given for $D=D_{i}$ and $D=D_{f}$. (a) $D_{i}=0.05$, $D_{f}=0.084$; (b) $D_{i}=0.25$, $D_{f}=0.35$; (c) $D_{i}=0.4$, $D_{f}=0.51$.

In Figure 4 and Figure 5, the porous material behavior was shown in various cases at every one of which the prescribed value of porosity *D* was held constant. In the framework of Gurson’s model the porosity (damage) can evolve as the plasticity develops, see Equation 13. Hence, it should be interesting to allow the damage, in the framework of Gurson’s model, to evolve from a certain initial value $D_{i}$ to a final value $D_{f}$ which is determined by the model when the loading is terminated. The resulting response can be compared with HFGMC prediction in which the volume fraction of the pores is equal to $D_{i}$ and $D_{f}$. Figure 6 presents the uniaxial stress response ${\bar{T}}_{22}-{\bar{F}}_{22}$ to a transverse loading of the porous material as predicted by Gurson’s and HFGMC model. In the Gurson model, initial porosity of $D_{i}=0.05,0.25$ and 0.4 evolve with loading to the final values: $D_{f}=0.084,0.35$ and 0.51, respectively at which the applied deformation is terminated at ${\bar{F}}_{22}=1.5$. The corresponding HFGMC predictions are shown in this figure for these values of $D_{i}$ and $D_{f}$. It can be readily observed that Gurson’s predictions extend between HFGMC prediction for $D=D_{i}$ and $D=D_{f}$.

**Figure 7:**
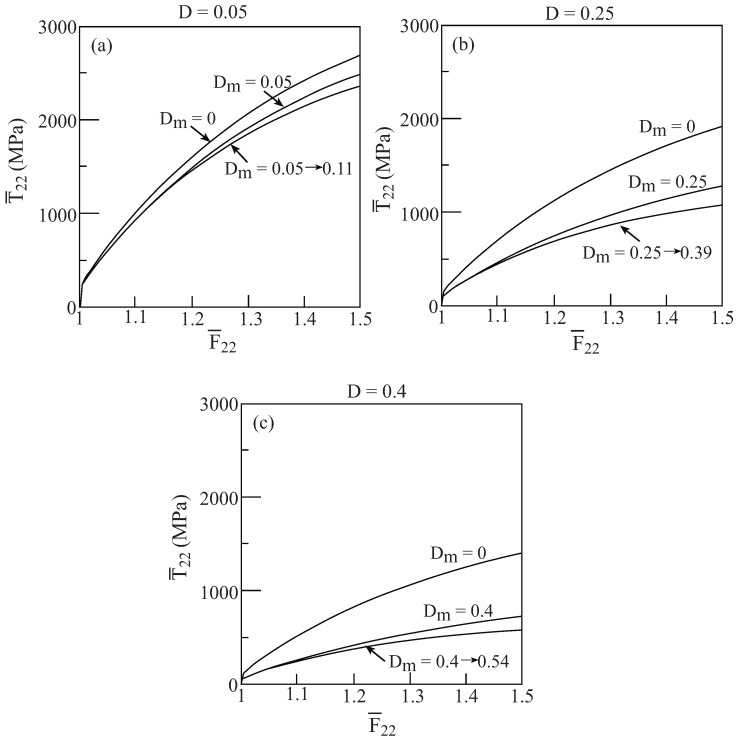
The response of a porous elastoplastic material modeled by Gurson’s equations (*D* is the amount of porosity) to a uniaxial stress loading applied in the transverse 2-direction. The properties of the matrix are given by Table 1. With $D_{m}$ denoting the amount of damage in the matrix, the response is given in the following three cases. (1) $D_{m}=0$, (2) $D_{m}=D$, and (3) $D_{mi}=D$ evolving to the final value $D_{mf}$ when ${\bar{F}}_{22}=1.5$. (a) D=0.05, (b) D=0.25, (c) D=0.4.

As a final investigation of the effect of evolving damage in the Gurson’s model, we consider again, in the framework of the HFGMC, a composite material whose matrix is characterized by Table 1 while the second phase is a pore. Let *D*, as before, denotes the amount of porosity of this composite. As the loading of the composite (porous) material increases, damage evolves in the ductile matrix the amount of which is denoted by $D_{m}$. Figure 7 shows the uniaxial stress response ${\bar{T}}_{22}-{\bar{F}}_{22}$ to a transverse loading of the composite whose porosity is *D* when: (1) $D_{m}=0$ (*i.e.*, no damage takes place in the matrix), (2) $D_{m}=D$ (*i.e.*, the damage in the matrix is kept equal to *D*) and, (3) $D_{m}$ evolves from $D_{mi}$ to the final value $D_{mf}$ when the loading reaches ${\bar{F}}_{22}=1.5$. In this last case and as indicated in Figure 7, for D=0.05, $D_{mi}=0.05$ reaching the final value of $D_{mf}=0.11$; for D=0.25, $D_{mi}=0.25$ reaching the final value of $D_{mf}=0.39$; and for D=0.4, $D_{mi}=0.4$ reaching the final value of $D_{mf}=0.54$. This figure well exhibits the effect of zero, constant and evolving damage in the ductile matrix of a porous material (composite) in which the amount of porosity is prescribed..

### 7.2. Application of the finite strain Lemaitre’s coupled elastoplastic-damage model

In the present subsection, a unidirectional composite that consists of a rubber-like hyperelastic matrix reinforced by ductile fibers whose properties are given in Table 1 is considered.

**Figure 8:**
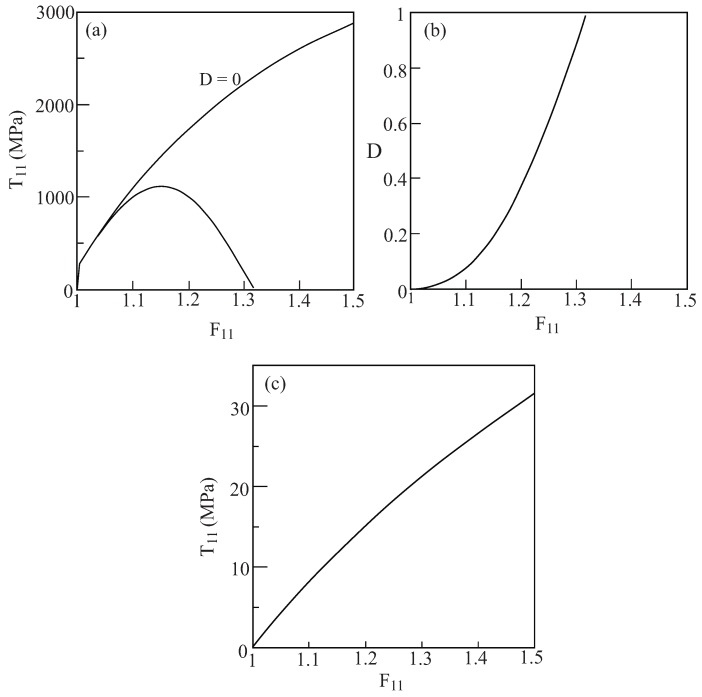
(a) The uniaxial stress response of the monolithic metallic material whose properties are given by Table 1. Its constitutive relations are given by the Lemaitre elastoplastic model. The response is shown in the absence (D=0) and evolving damage and, (b) the form of the damage evolution with the applied displacement gradient. (c) The uniaxial stress response of the monolithic hyperelastic matrix whose strain energy is given by Equation 79.

The metallic fibers are modeled by Lemaitre’s coupled elastoplastic-damage representation that was discussed in subsection 2.2 , where the values of the parameters *r* and *s* are: r=4.5MPa, s=1. Figure 8(a) shows the uniaxial stress response response of the monolithic ductile material in the absence of any damage (*i.e.*, D≡0) and the corresponding case in which the damage evolves from D=0 according to Equation 24. The response in the latter case is terminated when the damage approaches its final value D=1. The evolution of the damage is exhibited in Figure 8(b) which shows its gradual increase with the applied loading.

The rubber-like matrix is modeled as a hyperelastic compressible neo-Hookean material whose strain energy function is given by (Bonet and Wood [26])

(79) $$W=\frac{\mu }{2}\left(I_{1}-3-2\log J\right)+\frac{\lambda }{2}{\left(\log J\right)}^{2}$$

where $I_{1}$ is the first invariant of the right Cauchy-Green deformation tensor C, J=detF and *λ* and *μ* are material constants. The second Piola-kirchhoff stress tensor S is obtained from

(80) $$S=2\frac{\partial W}{\partial C}$$

and the constitutive relations of this material can be established in the form given by Equation 15, where the instantaneous first tangent tensor R of the material is given by

(81) $$R=4F\frac{\partial^{2}W}{\partial C\partial C}F^{T}+S⊗I$$

Figure 8(c) shows the uniaxial stress behavior of the monolithic hyperelastic material characterized by: λ=980MPa and μ=30MPa.

Consider this metal/rubber-like composite in which the continuous metallic fibers are oriented in the axial 1-direction. The volume fraction of the fibers is $v_{f}=0.1$. Let the composite be subjected to an off-axis uniaxial stress loading. Here, the fibers which are oriented in the 1-direction, are rotated around the 3-direction by an angle *ϕ*. As a result, a new system of coordinates (X,Y,Z) is obtained such that $Z=X_{3}$. The uniaxial stress loading is applied in the *X*-direction which is at angle *ϕ* with respect to the fibers direction. Referring to this new system of coordinates, the composite is loaded by the application of the deformation gradient $F_{XX}$, and all components of the first Piola-Kirchhoff stress tensor $T^{X}$, referred to the new coordinate system, are equal to zero except $T_{XX}$. In particular, $\phi =0^{\circ }$ and $90^{\circ }$ correspond to longitudinal and transverse uniaxial stress loading, respectively.

**Figure 9:**
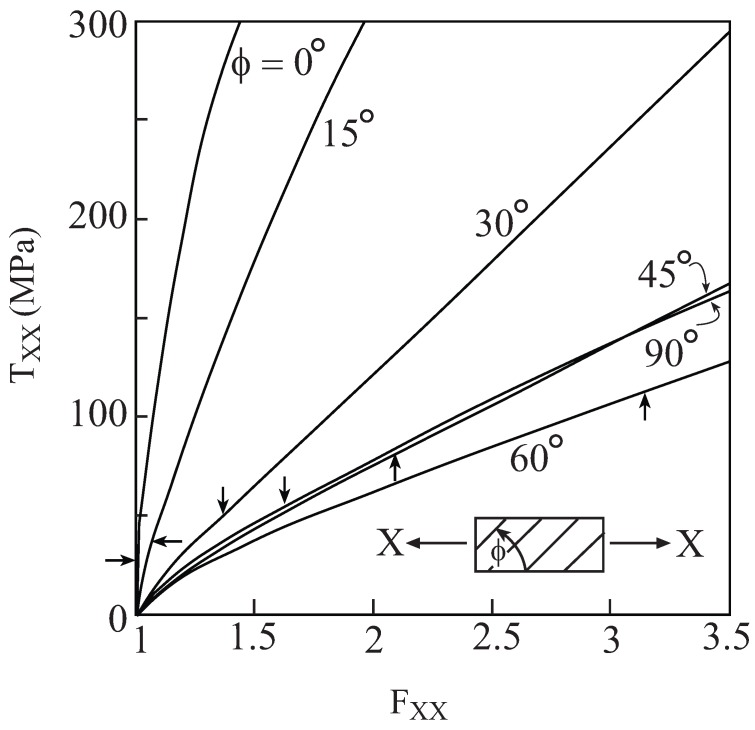
The response to uniaxial stress loading of a unidirectional metal/rubber-like composite in which no damage is taking place in the metallic phase (D≡0) which is modeled by Lemaitre elastoplastic equations. The rotation *ϕ* around the 3-direction denotes the angle between the fibers (oriented in the 1-direction) and loading (applied in the *X*-direction). The arrows indicate the yield stresses.

The locations of the initial yielding of this composite can be determined by generating its uniaxial stress response at various off-axis angles *ϕ* and detecting the stress at which yielding of the metallic fibers takes place. Figure 9 shows the off-axis stress-displacement gradient response of the composite in the absence of damage in the fiber phase (D≡0). The locations of yielding points are indicated by the arrows. It should be noted that the earliest yielding is obtained when the composite is loaded in the axial direction ($\phi =0^{\circ }$), whereas loading in the transverse direction ($\phi =90^{\circ }$) caused yielding at a later stage. Among the six off-axis angles at which the response of the composite is generated in the range of $1\leq F_{XX}\leq 3.5$, the highest yield stress is obtained at $\phi =60^{\circ }$.

**Figure 10:**
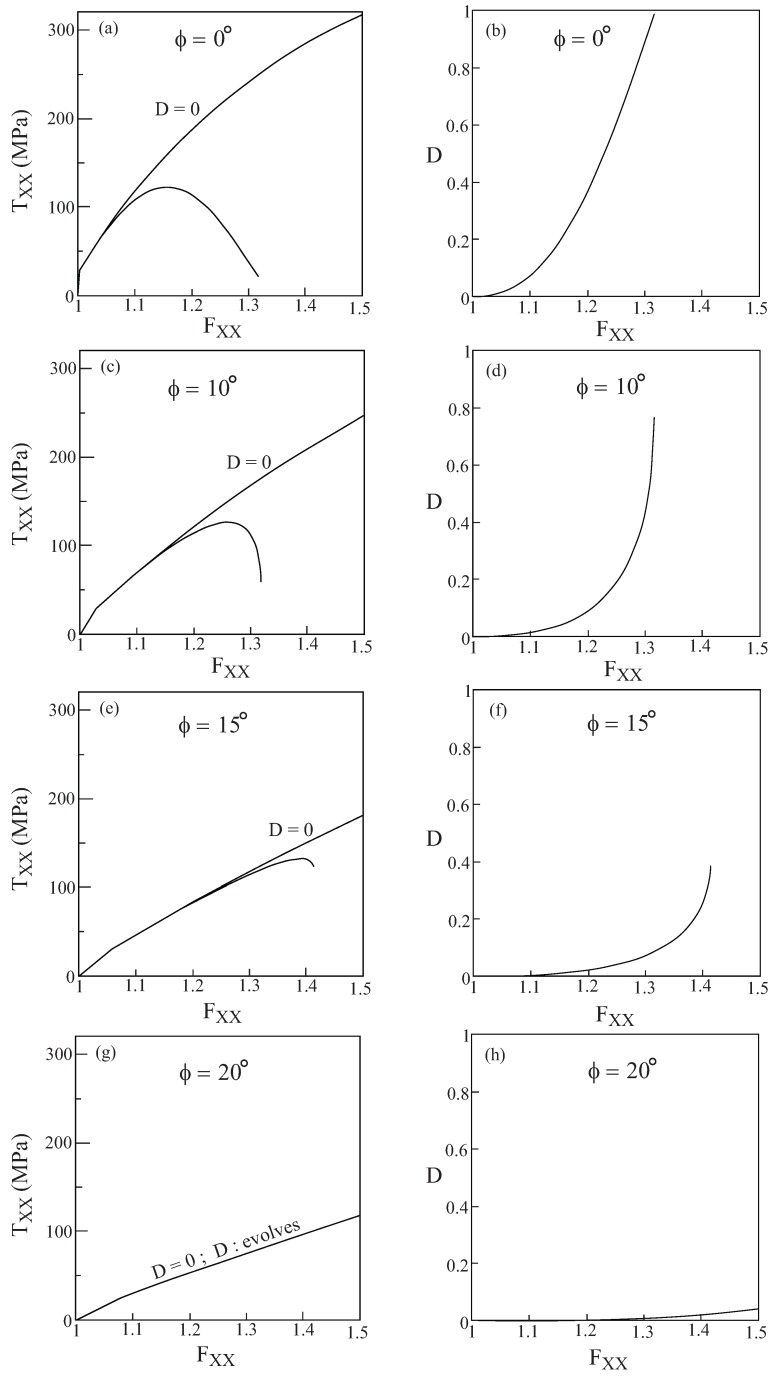
The response to uniaxial stress loading at various off-axis angles *ϕ* of a unidirectional metal/rubber-like composite in the absence (D=0) and presence of evolving damage in the fiber phase which is modeled by Lemaitre elastoplastic equations. Also shown in each case is the form of the damage evolution with applied deformation gradient. (a)-(b) $\phi =0^{\circ }$, (c)-(d) $\phi =10^{\circ }$, (e)-(f) $\phi =15^{\circ }$, (g)-(h) $\phi =20^{\circ }$.

Figure 10 presents that the metallic/rubber-like composite’s response to uniaxial stress loading at various off-axis angles. Here, both cases in which the damage in the fiber phase is absent D≡0 as well as when it is evolving from zero are shown. In the latter case, the dependence of damage evolutions on the applied deformation gradient are also shown. For loading in the axial direction $\phi =0^{\circ }$, the damage variable increases with applied loading until it reaches its final value of D=1. For the off-axis angle $\phi =20^{\circ }$, on the other hand, the amount of evolving damage in the fiber phase is very small, as a result of which the difference between the responses in the absence and presence of damage is indistinguishable. It should be noted that the for $\phi =0^{\circ }$, $10^{\circ }$ and $15^{\circ }$, the computations were terminated when the damage variables reached a certain value after which it jumped in the next step of computations to D=1.

**Figure 11:**
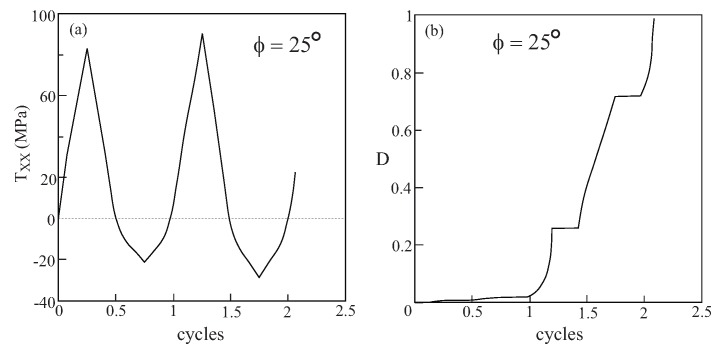
The response to uniaxial stress cyclic loading ($0.5\leq F_{XX}\leq 1.5$) at off-axis angle $\phi =25^{\circ }$ of a unidirectional metal/rubber-like composite in which the fibers are modeled by Lemaitre elastoplastic equations. (a) Variation of the stress $T_{XX}$ with the cycles, (b) the evolution of damage in the fiber phase.

It is possible of course to increase the amplitude of applied loading in Figure 10 above the value of $F_{XX}=1.5$ to get appreciable values of damage in the fiber constituent for the off-axis loading case $\phi =20^{\circ }$. It is interesting however to observe the response and damage evolution when the metallic/rubber-like composite is subjected to a cyclic loading such that $0.5\leq F_{XX}\leq 1.5$. Figure 11 presents the response of the composite and damage evolution in the fiber phase for a uniaxial stress cyclic loading for an off-axis angle of $\phi =25^{\circ }$. The figure clearly shows that the macroscopic stress $T_{XX}$ exhibits a cyclic behavior but the damage in the fiber constituent continue to increase while during unloading it retains its value. The figure indicates that the computations were terminated as the damage variable approached 1 after two cycles.

### 7.3. Application of the finite strain Gurson’s coupled viscoplastic-damage model

Let us consider a porous viscoplastic material in which the properties of its matrix are given in Table 1. This porous material is modeled by Gurson’s coupled viscoplastic-damage relations in conjunction with Equation 25 in which the viscosity and rate sensitivity parameters are: η=1s and ϵ=1, respectively. The applied rate of loading is $1s^{-1}$. As in the elastoplastic case, the resulting response of the considered viscoplastic porous material can be compared with the prediction obtained from HFGMC in which the bulk matrix is described by Table 1 in conjunction with the above parameters and rate of loading.

**Figure 12:**
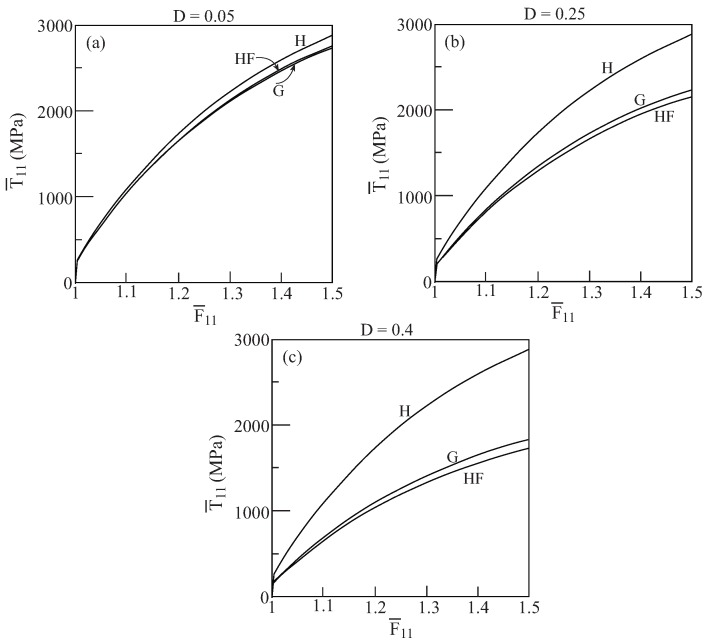
Comparisons between the uniaxial stress response to loading in the axial 1-direction of the porous viscoplastic material whose matrix properties are given in Table 1 as predicted by the Gurson (G) and HFGMC (HF) models. Also shown for a reference is the uniaxial stress response of the homogeneous viscoplastic material (H). (a) D=0.05, (b) D=0.25, (c) D=0.4.

**Figure 13:**
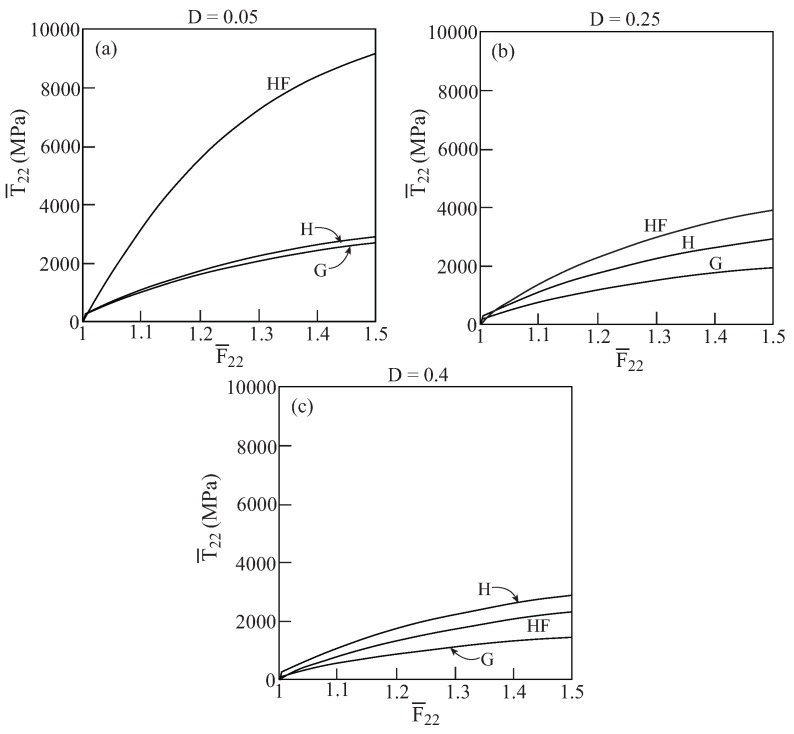
Comparisons between the uniaxial stress response to loading in the transverse 2-direction of the porous ductile viscoplastic material whose matrix properties are given in Table 1 as predicted by the Gurson (G) and HFGMC (HF) models. Also shown for a reference is the uniaxial stress response of the homogeneous viscoplastic material (H). (a) D=0.05, (b) D=0.25, (c) D=0.4.

Figure 12 exhibits the response of the viscoplastic porous material with various amount of porosities as predicted by the Gurson’s and HFGMC models. The porous material is uniaxially stress loaded in the axial 1-direction. Also shown is the corresponding behavior of the homogeneous viscoplastic material. In all cases there is no damage evolution. This figure is the viscoplastic counterpart of Figure 4. It should be noted that although the graphs of Figure 4 and Figure 12 exhibit similar behavior, the values of the responses are generally different. Indeed, for the axial loading in the 1-direction (along which the porosities are oriented) the predictions provided by the Gurson’s and HFGMC viscoplasticity are just like the time-independent elastoplastic case being quite close. However, for loading in the transverse 2-direction such closeness is not obtained. The responses in this case are shown in Figure 13 where the porous material is uniaxially stress loaded in the transverse direction. Note that the scale of the plots in Figure 13 is different from that of Figure 12 or of the elastoplastic counterpart that is shown by Figure 5. The largest difference between Gurson’s and HFGMC prediction is obtained for the lowest value of porosity (D=0.05) and it decreases rapidly with increasing porosity.

### 7.4. Application of the finite strain Lemaitre’s coupled viscoplastic-damage model

Consider the ductile material whose properties are given by Table 1. This material is represented by the Lemaitre’s coupled viscoplastic-damage model, see Equation 26, in which the viscosity and rate sensitivity parameters are: η=1s and ϵ=1, respectively. In all cases shown herein the loading was applied at a rate of $1s^{-1}$, and r=4.5MPa, s=1.

**Figure 14:**
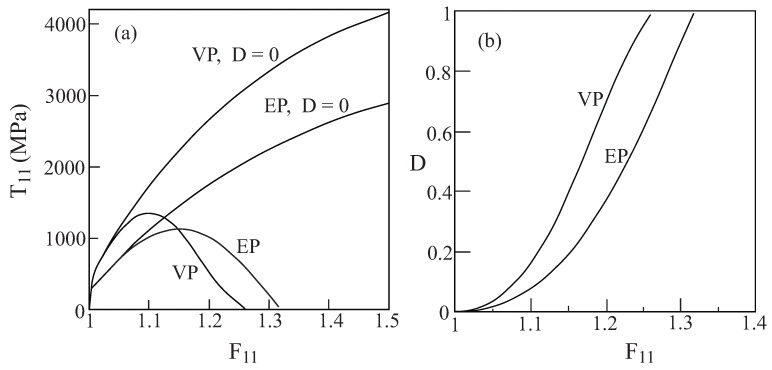
A comparison between the response and damage evolution of the monolithic ductile material, whose properties were specified by Table 1, when it is modeled by Lemaitre viscoplastic (VP) and elastoplastic (EP) equations. Also shown are the corresponding special cases when the damage is ignored (D≡0). (a) Stress-deformation response and, (b) damage evolution.

Figure 14 shows a comparison between the response of the homogeneous ductile material when it is represented by Lemaitre’s viscoplastic and elastoplastic coupled with damage models. The computations were terminated when the damage *D* approaches unity. This figure shows also the special case in which no damage takes place (*i.e.*, D≡0). As it is expected, the elastoplastic case exhibits lower stress values as compared to the viscoplastic behavior, but the evolution of damage is more rapid in the viscoplastic case.

**Figure 15:**
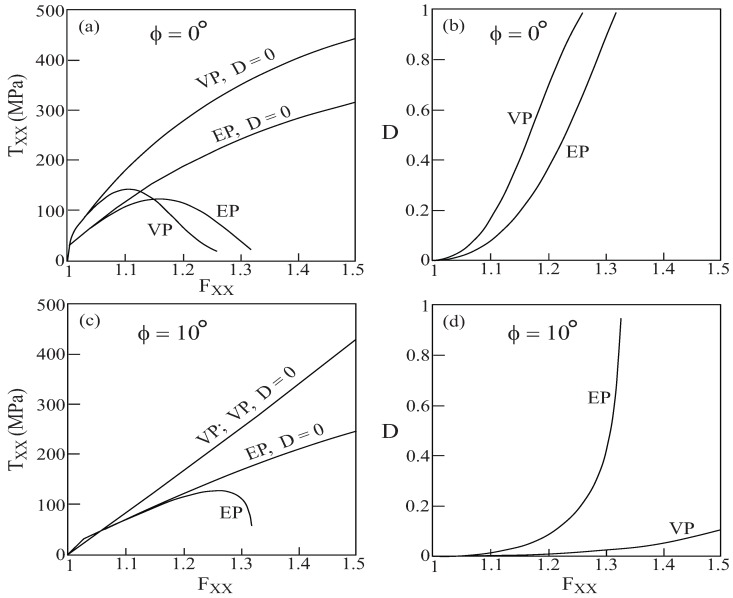
The response to uniaxial stress loading at two off-axis angles of a unidirectional metal/rubber-like composite in the absence (D=0) and presence of evolving damage in the fiber phase which is modeled by Lemaitre viscoplastic (VP) and elastoplastic (EP) equations. Also shown in each case is the form of the damage evolution with applied deformation gradient. (a)-(b) $\phi =0^{\circ }$, (c)-(d) $\phi =10^{\circ }$

The behavior of a unidirectional metal/rubber-like composite (with fiber volume fraction $v_{f}=0.1$) is shown in Figure 15 in which the metallic fibers (whose properties are given by Table 1) are represented by Lemaitre’s viscoplastic and elastoplastic coupled with damage models, whereas the rubber-like matrix behavior is given by Equation 79. The responses shown in this figure are caused by the application of uniaxial stress loadings $F_{XX}$ applied at off-axis angles $\phi =0^{\circ }$ and $10^{\circ }$ with respect to the fibers. The figure shows also the damage evolution in the metallic phase and the response in the special case in which the damage in the fibers is ignored. For the axial loading case ($\phi =0^{\circ }$) the elastoplastic and viscoplastic response and damage evolution are quite similar. The graphs show however that in contradistinction to the elastoplastic case (Figure 10c,d), damage evolution in the viscoplastic case when $\phi =10^{\circ }$ is relatively small as compared to the elastoplastic prediction in which the damage approaches unity. As a result, the stress-deformation curve in the viscoplastic case is indistinguishable from the corresponding one in which the damage is ignored.

**Figure 16:**
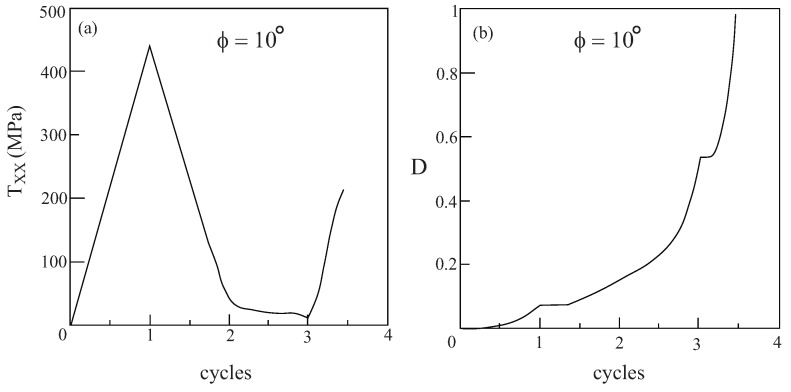
The response to uniaxial stress cyclic loading ($0.5\leq F_{XX}\leq 1.5$) at off-axis angle $\phi =10^{\circ }$ of a unidirectional metal/rubber-like composite in which the fibers are modeled by Lemaitre viscoplastic equations. (a) Variation of the stress $T_{XX}$ with the cycles, (b) the evolution of damage in the fiber phase.

As shown in Figure 15d, the evolution of damage in the metallic phase in the viscoplastic case is quite weak. Consequently, let the unidirectional metal/rubber-like composite be subjected to a uniaxial stress cyclic loading $0.5\leq F_{XX}\leq 1.5$ at the off-axis angle $\phi =10^{\circ }$. The resulting response of the composite and the damage evolution in the fiber phase are shown in Figure 16. The graph shows that the damage increases rapidly and total failure of the fiber occurs after less than 3.5 cycles.

### 7.5. Application of the finite strain coupled viscoelastic-damage model

**Figure 17:**
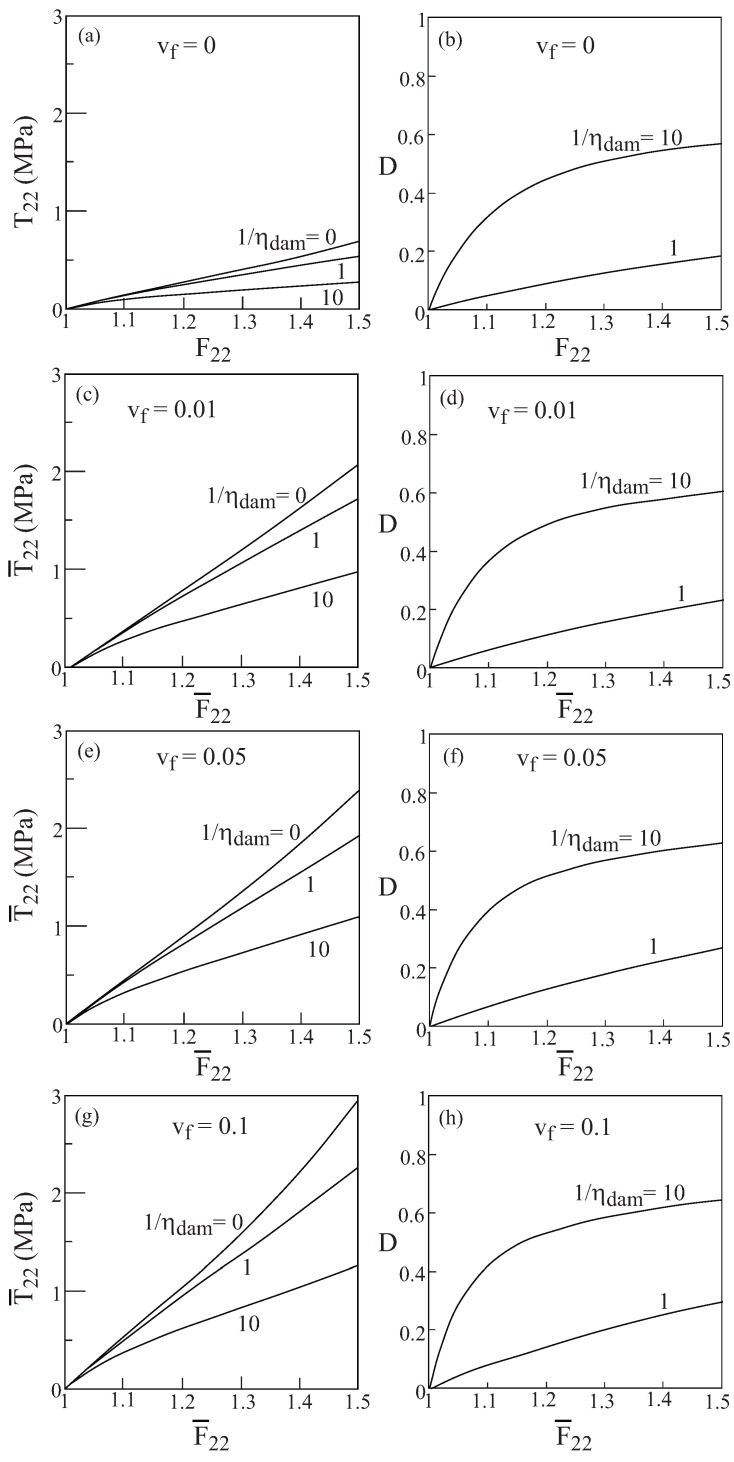
The response to transverse uniaxial stress loading applied at a rate of $1s^{-1}$ of a composite that consists of a finite viscoelastic matrix reinforced by nylon elastic fibers oriented in the axial 1-direction. The figure shows the stress response and the resulting damage evolution in the matrix for various values of fiber volume ratio $v_{f}$ and damage parameter $\eta_{dam}$. (a)-(b) Stress and damage evolution in the (unreinforced) matrix, (c)-(d), (e)-(f) and (g)-(h) stress and damage evolution for composites with fiber volume fraction $v_{f}=0.01,\;0.05$ and 0.1, respectively.

Let us consider a composite material that consists of a finite viscoelastic matrix whose free energy is given by Equation 44, which represents a single Maxwell element with the parameters given by Table 2. The damage parameters in Equation 53 are: $D_{0}^{\infty }=1$ and $\alpha_{dam}=1$. The viscoelastic matrix is reinforced by continuous linearly elastic isotropic nylon fibers oriented in the 1-direction, whose Young’s modulus and Poisson’s ratio are 2GPa and 0.4, respectively. The volume fraction of the fibers is denoted by $v_{f}$. Figure 17a shows the response of the monolithic finite viscoelastic material that is subjected to a uniaxial stress loading applied at a rate of $1s^{-1}$ for three values the damage parameter $1/\eta_{dam}=0,1,10$. Figure 17b exhibits the corresponding damage evolutions in these cases. Referring to Equation 51, the value of $1/\eta_{dam}=0$ corresponds to the case in which no damage takes place namely, D≡0. The other parts of Figure 17 shows the response of the unidirectional composite and the corresponding damage evolution in the matrix phase for various amounts of fiber volume ratio and the above three values of damage parameter $\eta_{dam}$. Changing the values of the latter parameters illustrates the effect of damage on the resulting behavior of the matrix and the overall response of the composite. The uniaxial stress loading is applied perpendicular to the fiber direction namely, in the transverse 2-direction. It should be noted that due to the large contrast between the moduli of the fiber and matrix, loading in the fiber direction would not exhibit the viscoelastic effects since in such a case the effect of the elastic fiber is dominant.

**Figure 18:**
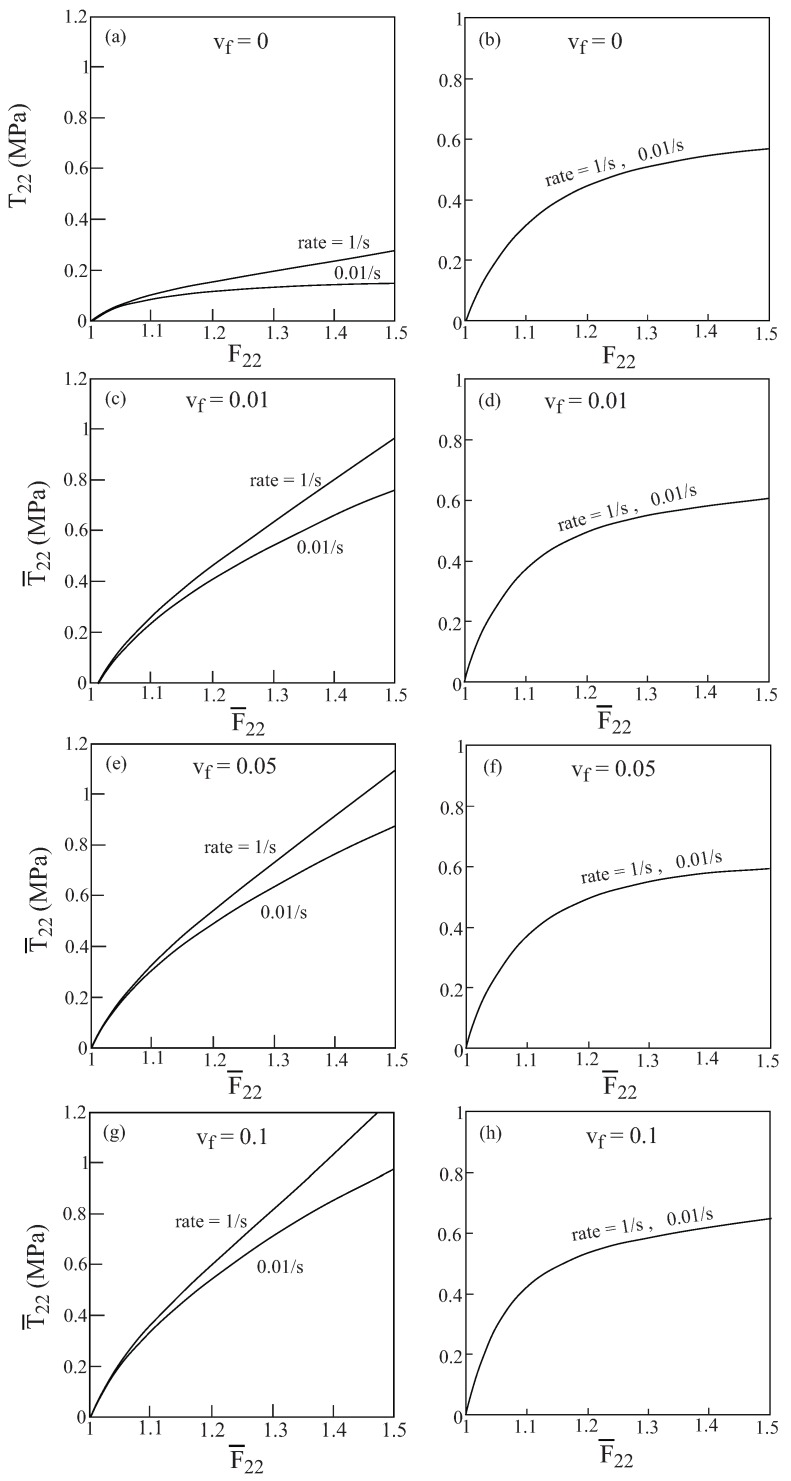
The response to transverse uniaxial stress loading applied at two rates of $1s^{-1}$ and $0.01s^{-1}$ of a composite that consists of a finite viscoelastic matrix reinforced by nylon elastic fibers oriented in the axial 1-direction. The figure shows the stress response and the resulting damage evolution in the matrix in which the damage parameter $\eta_{dam}=0.1$ is held constant for various values of fiber volume ratio $v_{f}$. (a)-(b) Stress and damage evolution in the (unreinforced) matrix, (c)-(d), (e)-(f) and (g)-(h) stress and damage evolution for composites with fiber volume fraction $v_{f}=0.01,\;0.05$ and 0.1, respectively.

The responses and damage evolutions in Figure 17 were caused by uniaxial transverse stress loading applied at a rate of $1s^{-1}$. Figure 18 exhibits the effect of applying the transverse loading at two different rates: $1s^{-1}$ and $0.01s^{-1}$ which is caused by the presence of the viscoelastic mechanism. In all cases shown in this figure, the damage parameter $\eta_{dam}=0.1$ is kept constant. Figure 18a,b show the response of the unreinforced finite viscoelastic matrix and the resulting damage evolution at these two rates, whereas the other portions of this figure exhibit the behavior of the nylon reinforced matrix with various fiber volume ratios. It is interesting to observe that whereas the stress responses are sensitive to the rate of applied loading, the damage evolution in the matrix is not sensitive in the sense that up to the scale of the plot the behaviors caused by these two rates are indistinguishable.

## 8. Conclusions and Future Research

A finite strain micromechanical analysis has been presented which is capable of predicting the behavior of multiphase materials that are modeled, in the framework of continuum damage mechanics, by finite elastoplasticity, viscoplasticity and viscoelasticity coupled with damage. Applications were presented in various circumstances. The present applications can be readily extended to obtain the finite strain response of laminated composites that are subjected to in-plane loading.

The two-dimensional macroscopic Gurson’s model which is suitable for the representation of porous materials, in which the porosity is oriented in the axial direction, was applied to both elastoplastic and viscoplastic phases and its predictions were compared with those provided by the HFGMC method. Extension to phases which are represented by the Gurson’s three-dimensional model, in which the porosity is given by a spherical pore, is a subject for a future research. In the present research, Gurson’s model was applied in its original form. The inclusion of damage threshold [12] according to which damage starts only at a critical value, and the incorporation of the improvements of Tvergaard [31]-[32] to obtain a closer agreement with numerical results of a periodic array of voids, and of Tvergaard and Needleman [33] to account for the effects of void nucleation and coalescence at failure is another subject for a future research.

In the present investigation, the finite strain constituent of the composite was modeled either as inelastic (time-dependent or time-independent) or viscoelastic constitutive relations. In Miehe and Keck [25] and Peric and Dettmer [30], finite strain generalized inelastic material models that combine elastic, inelastic and viscoelastic behavior was presented. Hence it is possible to generalize the present HFGMC model to include finite strain constituents in which the material behavior exhibits combined different rheological phenomena (elastic, elastoplastic, viscoplastic and viscoelastic). In the framework of infinitesimal strains, composites with phases that exhibit viscoelastic-viscoplastic behavior were investigated by Aboudi [34].

Due to the simplicity of the finite strain HFGMC model, it should not be difficult to link it to a finite element procedure in order to analyze composite structures (e.g., composite beams, plates and shells) undergoing large deformations. Indeed, the capability for such structural investigations has been already performed in the infinitesimal strain domain by Bednarcyk and Arnold [35] who presented a framework that enables coupled multiscale analysis of composite structures. To this end, they developed, free, finite element analysis-micromechanics analysis code (FEAMAC) software that couples the micromechanics analysis code of the generalized method of cells (MAC/GMC) with the commercial ABAQUS finite element software to perform micromechanics based finite element analysis such that the nonlinear composite material response at each integration point is modeled at each increment by MAC/GMC.

As to the small strain HFGMC method, It was recently coupled to ABAQUS software by Haj-Ali and Aboudi [36] to generate a nested local-global nonlinear finite element analysis of composite materials and structures. More recently, the hyperelastic HFGMC model has been coupled to the finite element ABAQUS software by Kim [37] to investigate the behavior of various types of tissue materials including the human arterial wall layers and porcine aortic valves leaflets. The results from this multiscale structural investigation were compared with the collagen fiber network (a model made of hyperelastic collagen and elastin layered finite elements) and Holzapfel *et al.* [38] and Gasser *et al.* [39] (hyperelastic anisotropic homogenized material) models. It was shown that the hyperelastic HFGMC is effective for the modeling of arteries especially when the collagen fiber network has a periodic microstructure.
